# Netrin-1 blockade inhibits tumour growth and EMT features in endometrial cancer

**DOI:** 10.1038/s41586-023-06367-z

**Published:** 2023-08-02

**Authors:** Philippe A. Cassier, Raul Navaridas, Melanie Bellina, Nicolas Rama, Benjamin Ducarouge, Hector Hernandez-Vargas, Jean-Pierre Delord, Justine Lengrand, Andrea Paradisi, Laurent Fattet, Gwenaële Garin, Hanane Gheit, Cecile Dalban, Ievgenia Pastushenko, David Neves, Remy Jelin, Nicolas Gadot, Nicolas Braissand, Sophie Léon, Cyril Degletagne, Xavier Matias-Guiu, Mojgan Devouassoux-Shisheboran, Eliane Mery-Lamarche, Justine Allard, Egor Zindy, Christine Decaestecker, Isabelle Salmon, David Perol, Xavi Dolcet, Isabelle Ray-Coquard, Cédric Blanpain, Agnès Bernet, Patrick Mehlen

**Affiliations:** 1 Centre Léon Bérard, Departement de Recherche Clinique, Centre de recherche en cancérologie de Lyon INSERM U1052-CNRS UMR5286, Université de Lyon, Université Claude Bernard Lyon1, Centre Léon Bérard, Lyon, France; 2 Basic Medical Sciences Department Oncological Pathology Group, Institut de Recerca Biomèdica de Lleida, Universidad de Lleida, Lleida, Spain; 3 Apoptosis, Cancer and Development Laboratory – Equipe labellisée ‘La Ligue’, LabEx DEVweCAN, Institut PLAsCAN, Centre de Recherche en Cancérologie de Lyon INSERM U1052-CNRS UMR5286, Université de Lyon, Université Claude Bernard Lyon1, Centre Léon Bérard, Lyon, France; 4 Netris Pharma, Lyon, France; 5 Centre de Recherche en Cancérologie de Lyon, INSERM U1052-CNRS UMR 5286, Centre Léon Bérard, Claude Bernard Lyon 1 University, Lyon, France; 6 Institut Claudius Regaud, IUCT-Oncopole, Toulouse, France; 7 Laboratory of Stem Cells and Cancer, WEL Research Institute, Université Libre de Bruxelles, Brussels, Belgium; 8 CRCL Core facilities, Centre de Recherche en Cancérologie de Lyon (CRCL) INSERM U1052-CNRS UMR5286, Université de Lyon, Université Claude Bernard Lyon1, Centre Léon Bérard, Lyon, France; 9 Hospices Civils de Lyon, Department of Pathology, Lyon, France; 10 Department of Pathology, IUCT-Oncopole, Toulouse, France; 11 DIAPath, Center for microscopy and molecular Imaging, Université Libre de Bruxelles, Gosselies, Belgium; 12 Laboratory of Image Synthesis and Analysis, Ecole Polytechnique-Université libre de Bruxelles, Brussels, Belgium; 13 Departement of Pathology, Erasme University Hospital, Université Libre de Bruxelles, Brussels, Belgium; 14 Centre Universitaire Inter Régional d’Expertise en Anatomie pathologique Hospitalière (CurePath), Jumet, Belgium

**Keywords:** Translational research, Tumour heterogeneity, Endometrial cancer

## Abstract

Netrin-1 is upregulated in cancers as a protumoural mechanism^1^. Here we describe netrin-1 upregulation in a majority of human endometrial carcinomas (ECs) and demonstrate that netrin-1 blockade, using an anti-netrin-1 antibody (NP137), is effective in reduction of tumour progression in an EC mouse model. We next examined the efficacy of NP137, as a first-in-class single agent, in a Phase I trial comprising 14 patients with advanced EC. As best response we observed 8 stable disease (8 out of 14, 57.1%) and 1 objective response as RECIST v.1.1 (partial response, 1 out of 14 (7.1%), 51.16% reduction in target lesions at 6 weeks and up to 54.65% reduction during the following 6 months). To evaluate the NP137 mechanism of action, mouse tumour gene profiling was performed, and we observed, in addition to cell death induction, that NP137 inhibited epithelial-to-mesenchymal transition (EMT). By performing bulk RNA sequencing (RNA-seq), spatial transcriptomics and single-cell RNA-seq on paired pre- and on-treatment biopsies from patients with EC from the NP137 trial, we noted a net reduction in tumour EMT. This was associated with changes in immune infiltrate and increased interactions between cancer cells and the tumour microenvironment. Given the importance of EMT in resistance to current standards of care^2^, we show in the EC mouse model that a combination of NP137 with carboplatin-paclitaxel outperformed carboplatin-paclitaxel alone. Our results identify netrin-1 blockade as a clinical strategy triggering both tumour debulking and EMT inhibition, thus potentially alleviating resistance to standard treatments.

## Abstract (web summary)

We describe netrin-1 upregulation in a majority of human endometrial carcinomas and demonstrate that netrin-1 blockade, using the anti-netrin-1 antibody NP137, is effective both in a mouse model and in patients with endometrial carcinomas.

## Main

Netrin-1 is an embryonic, secreted, laminin-related glycoprotein that plays key roles in neuronal navigation, angiogenesis and cell survival^1,3,4^. Netrin-1, which is expressed mainly during embryonic development, has been shown to be re-expressed by both cancer cells and the tumour microenvironment in a large proportion of human neoplasms^1,5^. Specifically this has been shown to occur in inflammation-associated colorectal cancer^6,7^, metastatic breast cancer^8^, lung cancer^9^, neuroblastoma^10^, lymphoma^11^ and melanoma^12^. In preclinical models mimicking these diseases, interference between netrin-1 and its receptors was sufficient to trigger cancer cell death and induce tumour growth inhibition^1,5,11,12^. Based on these findings, a monoclonal antibody (mAb) neutralizing netrin-1 and blocking the netrin-1–UNC5B interaction, dubbed NP137, was developed^13^ and underwent preliminary safety and efficacy assessment in patients with advanced solid tumours in a Phase 1 trial (NCT02977195). Owing to some objective responses observed in gynaecological cases during the dose-escalation phase, the extension phase of the trial was enriched in patients carrying endometrial tumours. A specific cohort has also been established, with mandatory biopsies before and after treatment, for translational research. Preliminary data from this trial were disclosed at the 2019 ESMO meeting^14^, but the trial is still ongoing, with patients continuing to receive treatment, and results will be fully reported after final analysis. In this article we report translational data generated in parallel with the Phase 1 study including patients with endometrial cancer and provide a series of preclinical and biopsy data demonstrating that NP137 not only reduces tumour cell number but also triggers inhibition of epithelial-to-mesenchymal transition (EMT) features, which ultimately increases tumour sensitivity to chemotherapy.

## Netrin-1 and endometrial adenocarcinomas

**Extended Data Fig. 1:**
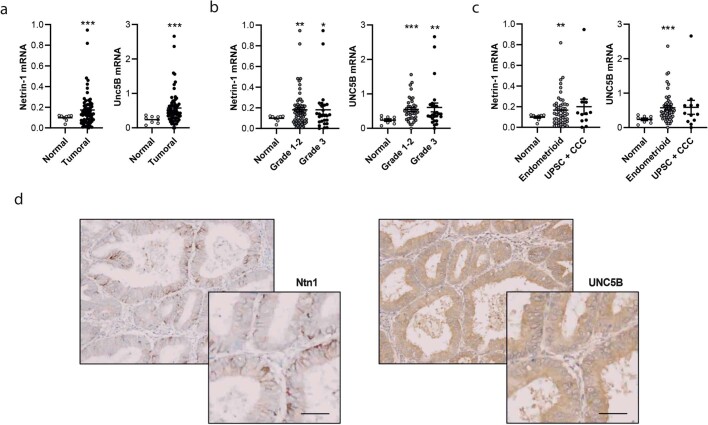
Netrin-1 and UNC5B are up-regulated in human endometrium adenocarcinoma. **a**, **b**, **c**, Relative mRNA expression of netrin-1 (left) and UNC5B (right) in patients with endometrial carcinoma (n = 73) compared to unpaired normal tissue (n = 8) (**a**) global expression (*** *P* < 0.001 by T-test one-sided) (**b**) expression reported to grades: Grade1+2 n = 38, Grade 3 n = 26, (** *P* = 0.002 and * *P* = 0.038, respectively normal vs grade 1-2 and grade 3 for Ntn1 and p < 0.001 and ** p = 0.004 for Unc5B by T-test one-sided) (**c**) expression reported to types: Endometrioid n = 51, UPSC+CCC n = 12 (UPSC: Uterine papillary serous carcinoma, CCC: Clear cell carcinoma) (** p = 0.002 and * p = 0.097, respectively normal vs endometrioid and UPSC+CCC for Ntn1 and *** p < 0.001 for Unc5B by T-test one-sided), defined by qRT-PCR (Bars are mean values mean ± s.e.m., data are normalized to TBP, PPIA and GUSB genes). **d**, Representative netrin-1 and UNC5B immunohistochemistry analysis of an endometrioid carcinoma (grade 1). 2x magnification field (bottom right). Scale bar (represented by a line,100 µm). Source data

We analysed netrin-1 expression by quantitative PCR with reverse transcription (RT–qPCR) in a cohort of 72 human endometrial tumours (Supplementary Table 1a). As shown in Extended Data Fig. 1a, netrin-1 is significantly upregulated in endometrial adenocarcinoma (EC) without specific change in grades or subtypes (Extended Data Fig. 1b,c). Its main receptor, UNC5B, was also found to be expressed more in tumour tissues than in normal endometrium (Extended Data Fig. 1a–c) while DCC, another netrin-1 receptor, is expressed neither in normal endometrium nor endometrial cancer. Netrin-1 (and UNC5B) positivity was monitored in most endometrial tumours by immunohistochemistry (IHC) (Extended Data Fig. 1d).

**Fig. 1:**
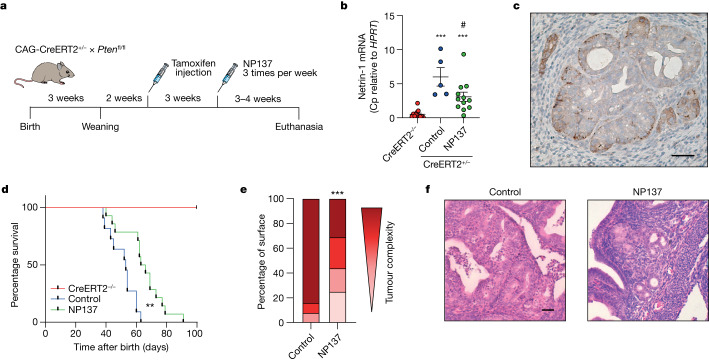
Netrin-1 blockade inhibits endometrial adenocarcinoma progression in preclinical models. **a**, Diagram showing the experimental strategy used to induce *Pten* deletion into CAG-CreERT2^+/−^*Pten* f/f mice using tamoxifen injection and either treatment with NP137 or control. Mice were euthanized if they experienced breathing difficulties^15^. **b**, Relative messenger RNA expression of netrin-1 as defined by RT–qPCR in the endometrium of CreERT2^–/–^ animals (*n* = 12) and in tumours induced following *Pten* deletion (tamoxifen injection) in CreERT2^+/−^ *Pten* f/f mice intraperitoneally treated with NP137 (10 mg kg^–1^) (*n* = 12) and in control (*n* = 5). Bars are mean ± s.e.m.; data normalized to *HPRT* gene; Cp, crossing point. ***P < 0.001 CreERT2^+/–^ versus CreERT2^–/–^ and ^#^*P* = 0.0284 NP137 versus control by Mann–Whitney two-sided test. **c**, Representative netrin-1 IHC analysis of EC in CreERT2^+/−^ *Pten* f/f mouse following 6 weeks of tamoxifen injection. Scale bar, 100 µm. **d**, Kaplan–Meier curves indicating percentage survival for normal mice (CreERT2^–/–^, red, *n* = 8), *Pten-*deleted mice treated with NP137 (green, *n* = 14) and control (blue, *n* = 11). **P < 0.01 by Mantel–Cox test. **e**, Quantification by pathologists of endometrial lesions, presented by tumour complexity (progressively darker colour from hyperplasia through mild, then moderate, endometrial intraepithelial neoplasia to adenocarcinoma) between control mice (*n* = 12) and those treated with NP137 mAb (*n* = 16). ***P < 0.001 by chi-squared test and Fisher’s two-sided exact test. **f**, Representative images of H&E staining of uterus from mice killed at week 6 of tamoxifen injection, those treated with NP137 and control. Scale bar, 50 µm. Source data

**Extended Data Fig. 2:**
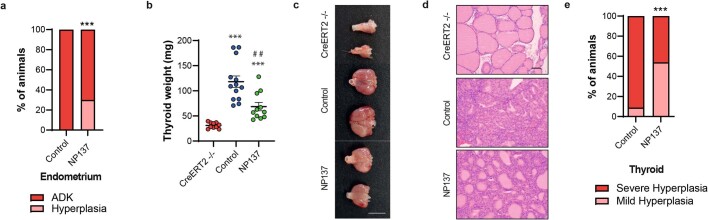
Netrin-1 blockade impacts tumor growth in a preclinical mouse model. **a**, Percentage of mice treated with control (n = 12) or NP137 (n = 16), showing hyperplasia or Adenocarcinoma (ADK) of the endometrium. *** *P* < 0.001 by Chi-square test and Fischer’s exact test. **b**, Quantification of the thyroid weight in CreERT2−/− mice (n = 10) or after *Pten* deletion (Tamoxifen injection) in CreERT2+/−x *Pten* f/f mice treated with control (n = 13) or NP137 (n = 11). Bars are mean values mean ± s.e.m., *** *P* < 0.001 against CreERT2−/−; ## *P* = 0.015 NP137 against control by Mann-Whitney two-sided. **c**, Representative images of the thyroids in panel **b**. **d**, Representative images of H&E staining of thyroid from mice sacrificed in weeks 5-6 after tamoxifen injection. Scale bar represented by a line, (100 µm) similar observations have been done on the full cohort. **e**, Percentage of mice showing mild or severe hyperplasia of the thyroid in mice treated (n = 16) or not with NP137 (n = 12). *** *P* < 0.001 by Chi-square test and Fischer’s exact test. Source data

We thus moved to a preclinical model recapitulating the development of EC, using the genetically engineered, temporally controlled *Pten* f/f-deleted mouse model (namely, the tamoxifen-inducible CAG-CreERT^+/−^ promoter), which has been shown to develop EC in situ rapidly, as well as thyroid hyperplasia^15^ (Fig. 1a). We thus treated control and *Pten*-deleted mice for 3–4 weeks with NP137 (10 mg kg^–1^, three times per week) and analysed netrin-1 expression and tumour progression. As shown in Fig. 1b,c, netrin-1 was upregulated in mouse tumours following deletion of *Pten*, and this upregulation was decreased following NP137 treatment. Of interest, NP137 was associated with decreased development of endometrial tumours (Extended Data Fig. 2a) and increased survival (Fig. 1d; note that there was no extension of the study after 7 weeks following *Pten* deletion because most mice showed breathing difficulties or other ailments due to development of thyroid tumours). Pathologists observed a decreased number of cancer cells following NP137 treatment (Fig. 1e) and healthier endometrial tissue (Fig. 1f). Similarly, antitumour activity was also observed in the thyroids of mice treated with NP137 (Extended Data Fig. 2b–e). These results indicate that targeting netrin-1 in EC inhibits tumour progression.

## Objective response in a patient with EC

**Fig. 2:**
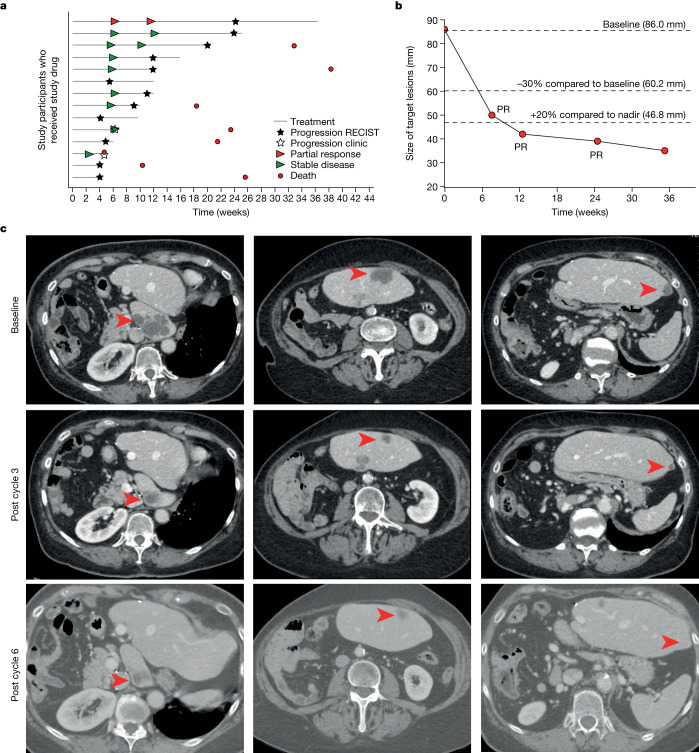
Clinical response in patients with EC following NP137 treatment. **a**–**c**, Fourteen patients (median age, 68.3 years (44.7–80.6); ECOG performance status 0, *n* = 5; ECOG performance status 1, *n* = 9) who had advanced or metastatic stage IV EC and were previously treated with a median of three (2.0–6.0) systemic treatment lines before inclusion were treated with NP137 (14 mg kg^–1^, *n* = 11 patients or 20 mg kg^–1^, *n* = 3 patients) with a median of 5.5 injections (2.0–17.0). **a**, Each bar represents one patient. Best responses to treatment are presented based on investigator review (according to protocol). Filled stars, radiological progression as per RECIST v.1.1; hollow stars, clinical progression as per investigator assessment; red arrowheads, partial response according to RECIST v.1.1; green arrowheads, stable disease according to RECIST v.1.1; red circles, death. **b**, Graph presenting the size evolution of target lesions (sum of two liver target lesions) from patient no. 02-004 treated intravenously with 14 mg kg^–1^ NP137 Q2W. Tumour response was assessed as partial response (PR) at 6 weeks and then at 3, 6 and 9 months; −30% reduction in target lesions size compared to baseline indicates partial response according to RECIST v.1.1. A dotted line showing the 20% increase in target lesions size compared to the nadir (minimum lesions size upon NP137 treatment) is also indicated. **c**, Abdominal transversal scans presenting liver metastasis at baseline, C3D1 (post cycle 3) and C6D1 (post cycle 6) from patient no. 02-004. Red arrowheads indicate lesions of interest. Source data

Based on previous results suggesting that netrin-1 blockade is a viable therapeutic strategy in cancer, a netrin-1-blocking antibody was developed for clinical use^13^ and is undergoing Phase I/II evaluation. We extracted efficacy data for 14 patients with EC from the ongoing Phase 1 study (Supplementary Table 1b,c). In this study, NP137 was administered once every 2 weeks (Q2W) as monotherapy until clinical/radiological progression. As shown in Fig. 2a and Extended Data Table 1a, no dose-limiting toxicity was observed and more than half of the patients (8 out of 14) had disease control (stable disease) as best response (best overall response, stable disease, 57.1%). In addition, a 74-year-old female patient with advanced EC had a RECIST1.1-defined partial response (patient no. 02-004; Supplementary Table 1b). The original diagnosis for this patient showed an endometrioid origin, a microsatellite stable phenotype, and expression of CK7, PAX8 and oestrogen receptors but no expression of CK20 or progesterone receptor. Before administration of NP137 she had received multiple therapeutic attempts, including adjuvant radiotherapy and carboplatin-paclitaxel followed by lurbinectedin as first-line treatment for metastatic disease, and a second attempt with carboplatin-paclitaxel, but had progression of liver metastases despite these therapeutics. A positron emission tomography–computed tomography (PET–CT) scan, performed at inclusion, confirmed intense uptake of fluorodeoxyglucose (before C1D1). She received 14 mg kg^–1^ intravenous NP137 Q2W and underwent a PET–CT scan at 6 weeks (that is, post cycle 3 of NP137), showing partial response according to RECIST v.1.1 with a 51% reduction in target liver lesions (Fig. 2b,c and Extended Data Table 1b). Partial response was confirmed on PET–CT scan at 3 months (Fig. 2c) and then again at 6 months, when size reduction of target lesions reached 55% (Extended Data Table 1b). This patient eventually experienced disease progression after 17 cycles of NP137 and went on to receive additional therapy, including letrozole A, immunotherapy and tamoxifen, without experiencing additional objective response.

## Netrin-1 blockade inhibits tumour EMT

**Fig. 3:**
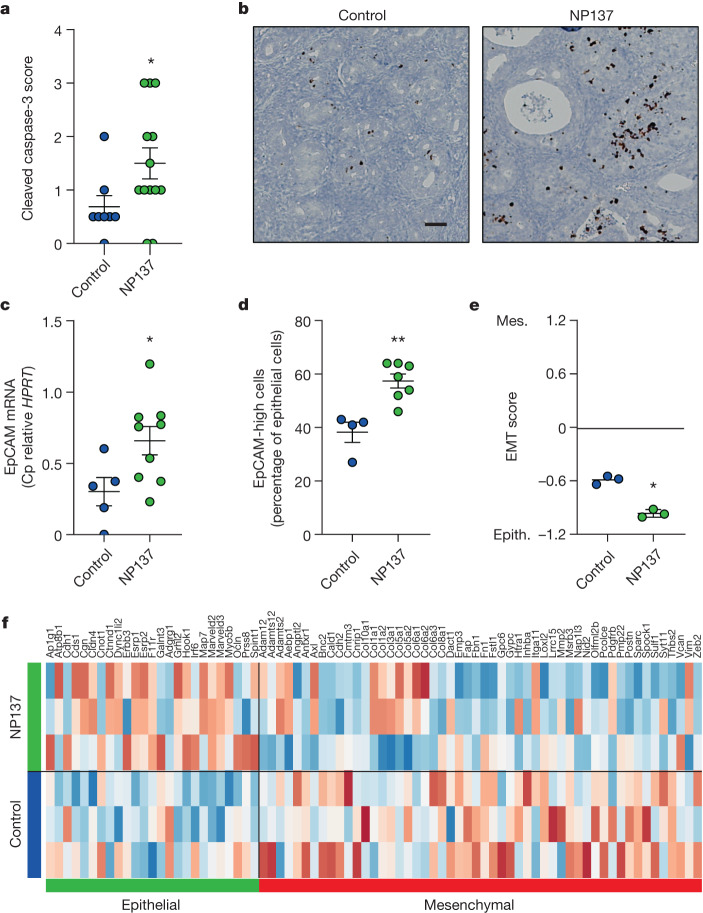
Netrin-1 blockade triggers apoptosis and EMT inhibition in a preclinical mouse model. **a**, Quantification of cell death using cleaved caspase-3 IHC in control (*n* = 8) and NP137 (*n* = 13)-treated tumours of CreERT2^+/−^*Pten* f/f mice. Bars are mean ± s.e.m.; *P = 0.0389 by Mann–Whitney two-sided test. **b**, Representative images of cleaved caspase-3 staining of **a**. Scale bar, 100 µm. **c**, Relative mRNA expression of EpCAM epithelial marker by RT–qPCR in mouse tumours, control (*n* = 5) and NP137 (*n* = 9). Bars are mean ± s.e.m., data normalized to *HPRT* gene; *P = 0.032 by Mann–Whitney two-sided test. **d**, Percentage of EpCAM high-expressing cells in control tumours (*n* = 4) versus NP137-treated (*n* = 7) as assessed by IHC. Bars are mean ± s.e.m.; **P = 0.0061 by Mann–Whitney two-sided test. **e**, EMT score (mouse orthologues of epithelial (epith.) or mesenchymal (mes.) signature from ref. ^20^) analysis derived from RNA-seq data, between control (*n* = 3) and NP137 (*n* = 3)-treated mice. Bars are mean ± s.e.m.; *P = 0.05 by Mann–Whitney one-sided test. **f**, Heatmap derived from RNA-seq data showing expression of EMT genes; control (*n* = 3) and NP137 (*n* = 3). Note that epithelial genes were upregulated under NP137-treated condition whereas mesenchymal genes were downregulated. Source data

To gain insight into the underlying mechanisms that link netrin-1 blockade to tumour growth inhibition we first analysed, based on the hypothesized mode of action of netrin-1 blockade, whether tumour growth inhibition was associated with tumour cell death in *Pten* f/f tumours treated with NP137. As shown in Fig. 3a,b, NP137 increased cancer apoptosis as measured by IHC on active caspase-3. We next performed RNA-seq of *Pten* f/f mouse tumours treated with NP137 versus untreated. Among the pathways/genes modulated following NP137 administration, we noted a decrease in EMT-related genes following treatment. Several in vitro studies have suggested the involvement of netrin-1 in EMT^16–19^, often associated with the PI3K/AKT pathway, which is frequently altered in endometrial cancer^17,18^. We then investigated whether NP137 might impact tumour EMT in the *Pten* f/f mouse model. We first assessed EpCAM epithelial marker expression in control versus NP137-treated tumours, and observed a statistically significant increase of this epithelial marker in NP137-treated tumours (Fig. 3c,d). To gain a more general view of tumour gene expression we then utilized a commonly used pancancer EMT signature^20^ that can be employed to determine EMT score^20^. We observed that treatment with NP137 decreased EMT score (Fig. 3e), associated with decreased expression of mesenchymal genes and increased expression of epithelial genes (Fig. 3f). These preclinical data support the view that netrin-1 blockade has a dual action on tumour cells: triggering of cancer cell death and inhibition of EMT features, rendering overall NP137-treated tumours more epithelial.

**Fig. 4:**
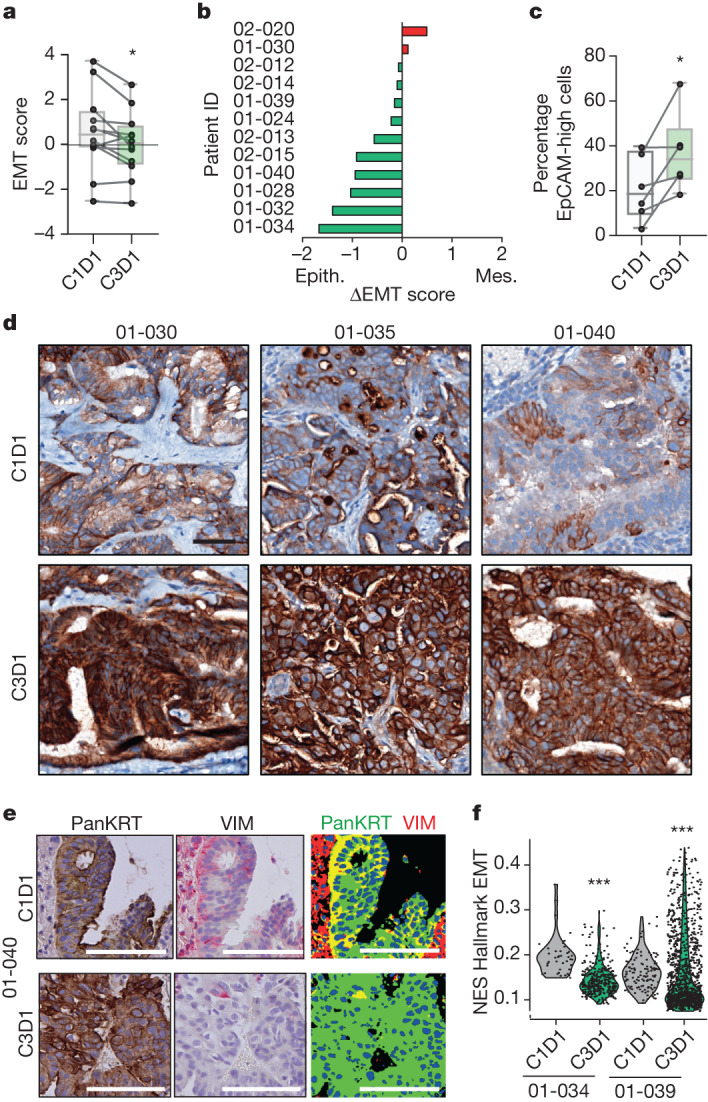
NP137 treatment inhibits EMT in patients with EC. **a**, Diagram showing EMT score calculated with Mak’s signature^20^ from RNA-seq of biopsies before (C1D1) and following two cycles of NP137 (C3D1) treatment (*n* = 12). Boxplots represent mean (25th–75th), whiskers range from minimum to maximum values and paired samples are identified on single-value representation; *P = 0.0161 by two-sided *t*-test. **b**, Swimmer plots showing individual evolution of EMT score for each patient; ΔEMT is the EMT score at C3D1 minus that at C1D1; ΔEMT < 0 means evolution towards epithelial phenotype (green) and >0 towards mesenchymal (red). **c**, Percentage of EpCAM high-expressing cells in C1D1 versus C3D1 biopsy samples as identified by IHC; *P = 0.0313 by Wilcoxon two-sided test (*n* = 6 patients). Boxplots represent mean (25th–75th), whiskers range from minimum to maximum values and paired samples are identified on single-value representation. **d**, Representative IHC of EpCAM in tumours in C1D1 and C3D1 for patient nos. 01-030, 01-035 and 01-040. Scale bar, 50 µm. **e**, Representative images of pancytokeratin (PanKRT) and vimentin (VIM) expression (colocalization of pancytokeratin (green) and vimentin (red) in the merged picture, right) in primary endometrial adenocarcinoma from patient no. 01-040 before and after NP137 treatment. Scale bars, 50 μm. Quantifications were performed on the full slides and similar results were observed for patient nos. 01-030 and 01-034. **f**, Analysis of tumour cell compartment in patient nos. 01-034 and 01-039 by Visium spatial gene expression. Violin plot of EMT UCell normalized enrichment score (NES) from tumoural histologically selected Visium spot between cells of C1D1 and C3D1 biopsy. ***P < 0.01 by Mann–Whitney two-sided test. Source data

We next determined whether the inhibition of EMT features observed in the preclinical models also occurs in patients treated with NP137. In the NP137 Phase 1 trial, among patients with EC described above, paired biopsies were collected at inclusion (C1D1) and after 1 month of treatment with the anti-netrin-1 compound (that is, just before the third infusion of NP137 (C3D1)). Bulk RNA-seq was successfully performed on 12 paired pre- and on-treatment biopsies (Supplementary Table 1b) and, as shown in Fig. 4a,b, two injections of NP137 were found sufficient to trigger a significant decrease in the pancancer EMT score described above, indicating an overall more epithelial phenotype of the tumour in patients after 1 month of treatment with NP137. The shift toward a more epithelial phenotype was confirmed by increased EpCAM IHC staining in tumour samples from patients treated with NP137 (Fig. 4c,d). We also observed a decrease in the proportion of tumour cells coexpressing pancytokeratin and vimentin (Fig. 4e).

**Extended Data Fig. 3:**
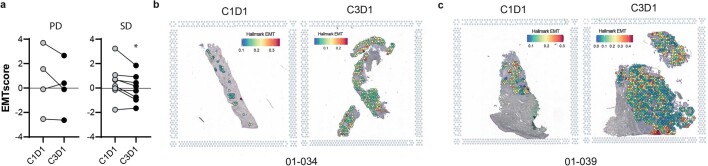
Pre/post biopsies analysis by bulk RNAseq or Visium Spatial Gene Expression. **a**. Diagram showing EMT-score calculated with the Mak’s signature^20^ as in Fig. 4a. Patients are segregated in PD (progression at 6 weeks) and SD (stable disease at least 6 weeks) according to their clinical benefit determined upon centralized review. * *P* = 0.0023; by T-Test two-sided. **b**, **c**, H&E-stained tissue from C1D1 (left panel) and C3D1 (right panel) with EMT score enrichment on Visium tumoral spot by UCell method. Source data

Of interest, when analysing changes in EMT score between baseline and on-treatment biopsies we observed that samples from patients who had disease progression as their best response on NP137 (progressive disease at first evaluation at 6 weeks) failed to show variation in EMT score. Conversely, there was a statistically significant decrease in EMT score in samples from patients who had disease control (at least stable disease at 6 weeks; Extended Data Fig. 3a).

To demonstrate more formally that the bulk change in EMT score seen with NP137 treatment in patients was occurring specifically in cancer cells, as a first approach we used the 10x Visium spatial gene expression system compatible with formalin-fixed, paraffin-embedded (FFPE) tissues. Two pairs of C1D1/C3D1 FFPE sections from two patients with EC were analysed (Extended Data Fig. 3b,c) and spatial gene expression profiling was performed in pathologist-selected regions of interest where only cancer cells could be identified by haematoxylin and eosin (H&E) staining. In both cases, EMT score was strongly decreased at C3D1 compared with C1D1 (Fig. 4f). Thus in patients, after 1 month of treatment with NP137, remaining cancer cells showed decreased EMT features compared with their pretreatment status.

**Fig. 5:**
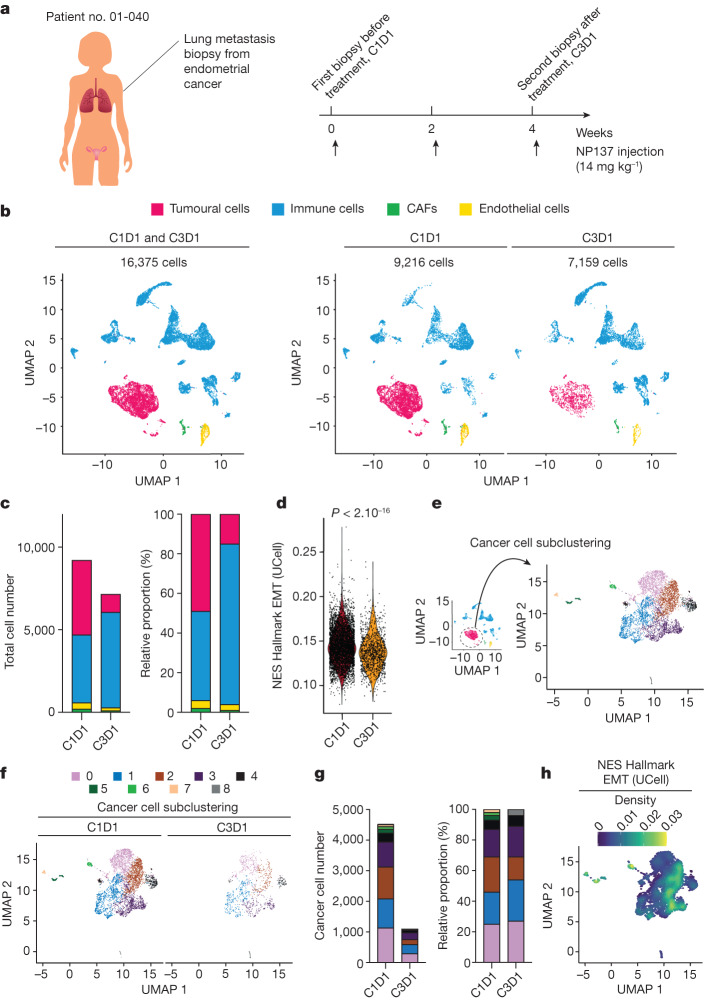
Single-cell RNA-seq analysis pre and post biopsy of a patient with EC. **a**, Illustration of patient no. 01-040 with two lung metastasis biopsies—one before treatment (C1D1), and one after two cycles of NP137 treatment (C3D1). **b**, Uniform manifold approximation and projection (UMAP) plot of 16,375 cells from two lung metastasis biopsies (left) or before treatment with 9,216 cells (C1D1, middle) and after treatment with 7,159 cells (C3D1, right), coloured by their four major cell types. **c**, Composition of major cell types in C1D1 and C3D1 biopsies. Left, total cell numbers in each condition; right, proportion of cells in each sample (note that cancer cell number decreased markedly after treatment). Colour coding as in **b**. **d**, Violin plot of EMT UCell enrichment score between cells of C1D1 and C3D1 biopsy (two-sided Wilcoxon test, *P* < 2.10^−16^). **e**, UMAP plot of subclustered cancer cells from the whole integrated dataset (C1D1 + C3D1). **f**, UMAP plot of subclustered cancer cells before and after treatment. **g**, Composition of cancer cell clusters in C1D1 and C3D1 biopsies. Left, cell numbers; right, proportion of cells in each sample (note that cancer cell number decreased markedly after treatment). **h**, Density plot of EMT UCell enrichment score showing clusters 2/3 with strong EMT enrichment (note that cluster 2 decreased after treatment (**g**, right)).

**Extended Data Fig. 4:**
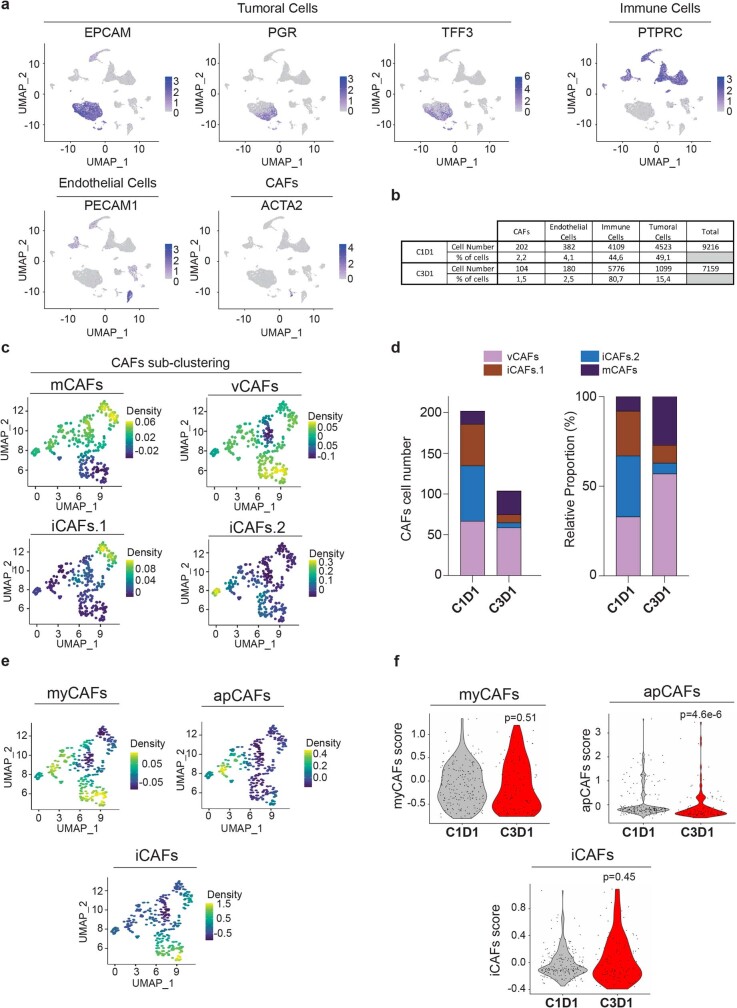
Pre/post biopsies scRNAseq identify global cell types and cancer cell sub-clustering. **a**, UMAP plot with the 2 integrated samples of patient 01-040 showing the normalized expression of major cell type markers: immune cells (*PTPRC*, also known as *CD45*), endothelial cells (*PECAM*), tumor cells (*EPCAM*, *PGR*, *TFF3*), CAF cells (*ACTA2*). **b**, Table showing the cell number in each cell type. **c**, Identification of CAFs subtypes (markers used: mCAFs: MMP2, DCN, COL12A1, FBLN1; vCAFs : MCAM, COL4A1, COL18A1; iCAFs.1: MUSTN1, TAGLN, S100A4, CXCL2; iCAFs.2: S100A8, CXCL8, SPLI). **d**, CAFs subcluster composition analysis, the left panel shows the total CAFs numbers *per* cluster in each condition and the right panel shows the proportion of CAFs *per* cluster in each samples. **e**, UMAP plot of sub-clustered CAFs showing other CAFs subtypes (markers used: myCAFs: ACTA2, CTGF, POSTN, PDGFR, TGFB1, COL1; iCAFs : IL6, CXCL1, CCL2, PDGFRA, HAS1, FAP, IL11, LIF; apCAFs: H2-AB1, CD74, SAA3). **f**, Violin plot showing CAFs subtypes scores before and after treatment (C1D1 or C3D1) (Wilcoxon test, two-sided).

To extend these studies to the single-cell level we performed 3′ single-cell RNA-seq (10x Genomics Next GEM 3′ kit) directly on fresh biopsies obtained at inclusion (C1D1, 9,216 cells) in the NP137 trial and after 1 month of treatment (C3D1, 7,159 cells) from a patient with advanced EC (patient no. 01-040; Supplementary Table 1b and Fig. 5a). Unsupervised clustering showed the presence of different cell populations including tumour cells (expressing EpCAM, PGR and TFF3—all markers of ECs^21^), immune cells (marked by CD45 (PTPRC) expression), cancer-associated fibroblasts (CAFs, marked by αSMA (ACTA2)) and endothelial cells (PECAM1 positive) (Fig. 5b and Extended Data Fig. 4a,b).

Treatment with NP137 led to a statistically significant decrease in the tumour cell compartment (Fig. 5b,c and Extended Data Fig. 4b). The proportion of tumour cells following anti-netrin-1 treatment was 3.19 times lower after two cycles of NP137 (Extended Data Fig. 4b). Of interest, in addition to the net decrease in cancer cells we noted a statistically significant decrease in EMT score in the whole tumour compartment, indicating an overall more epithelial phenotype associated with NP137 treatment (Fig. 5d). We also performed unbiased differential expression and pathway analysis only in tumour cells (Extended Data Table 2). Remarkably, EMT was the most significant term, with downregulation following exposure to NP137 (Extended Data Table 2). Of interest, while following NP137 treatment, most of the subcompartments of tumour cells decreased in a similar way and we noted that tumour cluster 2, which had the strongest decrease, also showed a high EMT score (Fig. 5e–h; note that clusters 5–7, with high EMT score, were not detectable following NP137 treatment but we cannot exclude the possibility that they were not captured, because these clusters already had very few cells at C1D1).

**Fig. 6:**
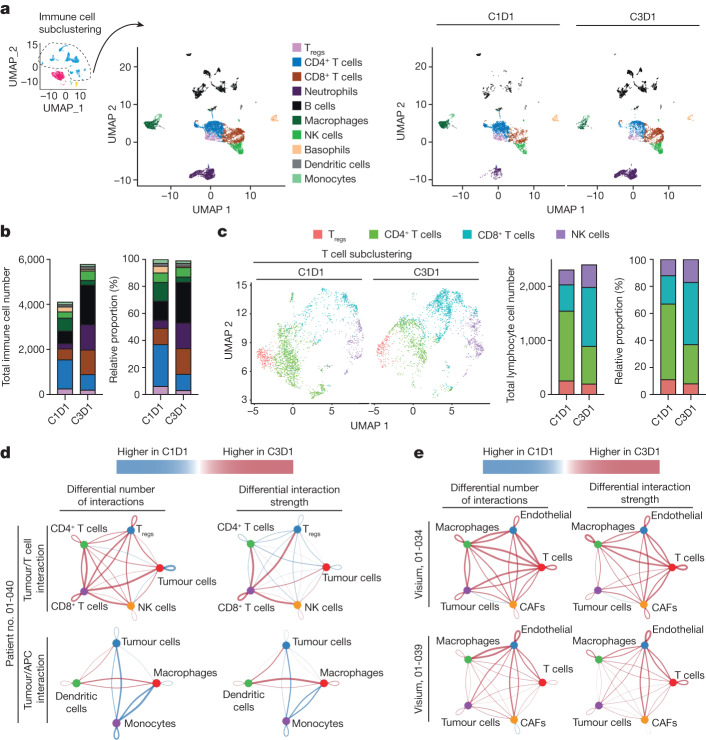
Immunological changes observed from single-cell RNA-seq analysis pre and post biopsy of a patient with endometrial adenocarcinoma. **a**, UMAP plots of subclustered immune cells from the whole integrated dataset (C1D1 + C3D1) for patient no. 01-040, illustrating the composition of major immune cell clusters in C1D1/C3D1 biopsies (left) or separately for C1D1 and C3D1 (right). **b**, Number (left) and proportion (right) of immune cell types in each sample. **c**, Left, UMAP plot of subclustered lymphocyte/NK cells from the whole integrated dataset (C1D1 and C3D1). T regulatory cells (T_regs_) were determined according to the markers shown in Extended Data Fig. 5b. Middle, right,composition of T/NK cell clusters in C1D1 and C3D1 biopsies; cell number (middle) and proportion of cells in each sample (right). Note that cytotoxic CD8^+^ T cell number increased after treatment whereas that of CD4^+^ cells decreased. **d**, CellChat analysis of single-cell assay in patient no. 01-040, showing the differential (C3D1/C1D1) number of interactions (left) and strength of interactions (right) between tumour cells and lymphocytes (top) and between tumour cells and APCs (bottom). **e**, CellChat analysis of Visium assay on patient nos. 01-034 (top) and 01-039 (bottom), showing the differential (C3D1/C1D1) number of interactions (left) and strength of interactions (right) between tumour cells and stromal cells. Line colours indicate higher numbers or strength interactions in C3D1 (red) and C1D1 (blue). Segment size is proportional to the difference in the number or strength of interactions between C3D1 and C1D1.

**Extended Data Fig. 5:**
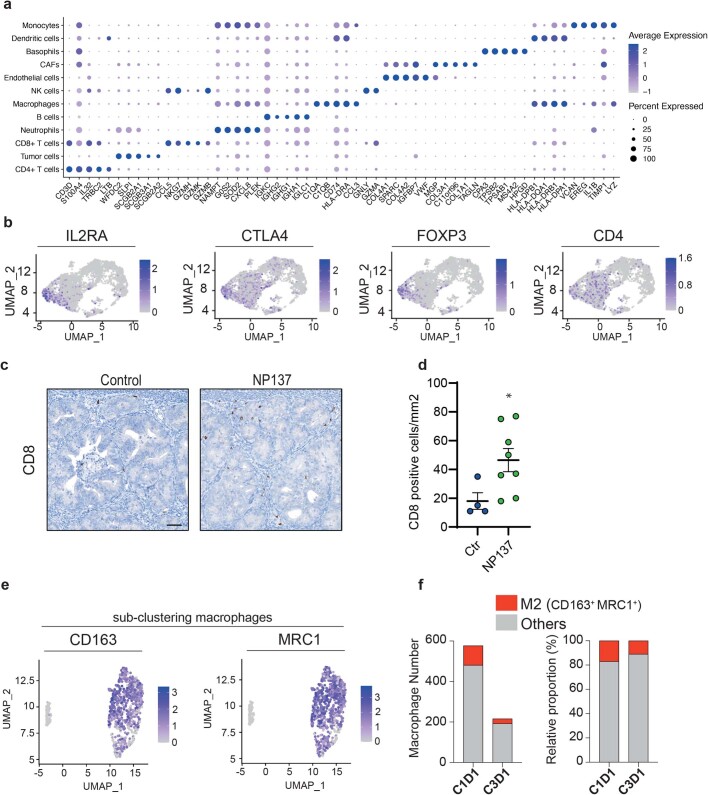
Pre/post biopsies scRNAseq identify various immune cell types. **a**, Validation of the cluster annotation with dot plot showing top 5 genes markers that are enriched in clusters which were previously characterized by automatic cell annotation package. **b**, Tregs cluster identification with UMAP plot of sub-clustered lymphocyte showing Tregs expression markers: IL2RA, CTLA4, FOXP3 and CD4. **c**,**d** CD8 detection and quantification by IHC in mouse *Pten* driven tumors treated with control or NP137. **c**, representative image of IHC. Scale bar = 50 µm. **d**, Quantification of CD8 positive cells in control tumors (n = 4) vs NP137 treated (n = 8). Bars are mean values mean ± s.e.m., * *P* = 0.0162 by Mann-Whitney two-sided. **e**, Analysis of M2-macrophages, we used *CD163* and *MRC1* as markers on the UMAP plot of sub-clustered macrophages with the 2 integrated samples. **f**, The 2 panels show the M2 cell type number and the proportion of M2 in each sample compared to the total macrophage number. Source data

**Extended Data Fig. 6:**
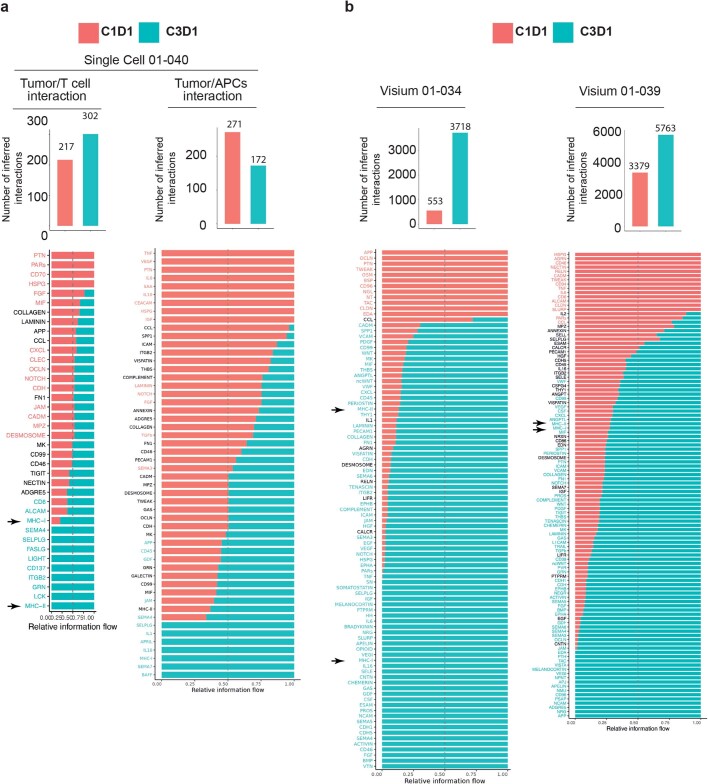
NP137 treatment impacts tumor cell communication with immune cell types. **a**, Histograms showing the number of inferred interaction (upper panel) from the single cell RNA seq analysis (patient 01-040) by CellChat packages and (lower panel) the significant genes were ranked based on their differences of overall information flow between C1D1 and C3D1. The left panels investigate the communication between Tumor and lymphocyte cells. The right panels investigate the communication between Tumor and APCs. **b**, Histograms showing the number of inferred interaction (upper panel) from the Visium assays (patients 01-034 and 01-039) by CellChat packages and (lower panel) the significant genes were ranked based on their differences of overall information flow between C1D1 and C3D1. The left and right panels correspond respectively to patient 01-034 and 01-039. Red and green are more enriched in C1D1 and C3D1 sample, respectively. Arrows indicate MHC-I and II genes which expression is enriched in C3D1 samples.

The change occurring in the tumoural compartment was also associated with a change in stromal cells (Figs. 5b,c and 6 and Extended Data Figs. 4c–f,  5 and 6a). Of note, NP137 treatment clearly appeared to have an impact on immune cells (Fig. 6a,b and Extended Data Fig. 5a). More specifically, following NP137 treatment we noted an increase in lymphocytes endowed with cytotoxic functions (CD8^+^ T cells and natural killer (NK) cells; Fig. 6c and Extended Data Fig. 5b). A similar increase in CD8^+^ cells was also noted in *Pten* f/f mice treated with NP137 (Extended Data Fig. 5c,d). Of interest, following NP137 treatment we noted both in the single-cell analysis from patient no. 01-40 and in the spatial transcriptomic data from patient nos. 01-034 and 01-039 an increase in both the number and strength of interaction between T cells and tumour cells (Fig. 6d,e). In particular, single-cell RNA-seq data showed an increase in the number and strength of interactions between CD8^+^ T cells and tumour cells (Fig. 6d). A decrease in M2 macrophages was detected (Extended Data Fig. 5e,f), together with an increase in major histocompatibility class I/II antigen presentation (Extended Data Fig. 6a,b). NP137 treatment is associated with more efficient antigen-presenting cells (APCs) because we observed a clear switch from monocytes in C1D1 to dendritic cells in C3D1 interacting with cancer cells (Fig. 6d,e and Extended Data Fig. 6b).

## NP137 inhibits chemotherapy resistance

**Extended Data Fig. 7:**
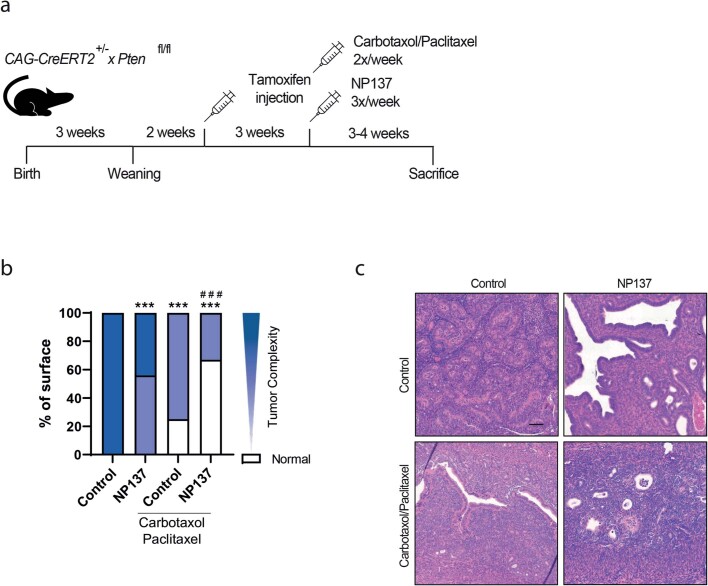
Combining NP137 and chemotherapy in *Pten* mutant mice. **a**, Diagram showing the experimental strategy to combine NP137 (10 mg/Kg, I.P. 3x/week) and Carboplatin Paclitaxel (respectively 30 mg/Kg and 15 mg/Kg, I.P. 2x/week) treatments in CAG-CreERT2+/−*Pten* f/f mice. **b**, Quantification by pathologists of the endometrial lesions presented by tumor complexities (progressively darker color from normal through hyperplasia, slight EIN (Endometrial intraepithelial neoplasia), moderate EIN and to adenocarcinoma) between mice treated with control (n = 6) or NP137 Ab (n = 9) alone or in combination with Carboplatin and Paclitaxel (n = 4 and 6). *** *P* < 0.001 against control alone; ###*P* < 0.001 against control with chemotherapy by Chi-square test and Fischer’s exact test. **c**, Representative H&E staining pictures of uterus from mice sacrificed in weeks 5-6 after tamoxifen injection, treated with control or NP137 alone or in combination with chemotherapy. Scale bar (represented by a line, 100 µm). Source data

Because of a large body of literature describing EMT as a major cause of chemotherapy resistance^2,22^ and because our data suggest that targeting of netrin-1 inhibits EMT, we assessed whether the addition of NP137 to carboplatin-paclitaxel (carbotaxol), the standard-of-care chemotherapy used in clinical settings in endometrial cancer, might enhance the efficacy of carbotaxol alone in the *Pten* f/f mouse model of endometrial adenocarcinoma. As shown in Extended Data Fig. 7, NP137/carbotaxol treatment proved superior to carbotaxol alone, leading to complete responses in mice. Altogether, these preclinical and clinical data demonstrate that a clinical-stage drug both induces tumour cell debulking and triggers a general inhibition of EMT features, which offers the possibility of alleviating resistance to conventional therapies.

## Discussion

We provide here documentation of a clinical activity of netrin-1 titration using NP137 as monotherapy. Whereas the interest in targeting netrin-1 in the cancer field is relatively recent^13^, this report indicates that targeting netrin-1 in endometrial cancer may be effective. In addition to the generation of a series of data, including analysis of a human cohort and preclinical experiments in mice, we have confirmed a key mechanistic role for netrin-1 in endometrial cancer resistance and progression. We also report a partial response detected in a patient with EC receiving an anti-netrin-1 mAb. This partial response in a human patient, together with decrease in tumour cell counts in mice and marked decrease in tumour cells observed in single-cell RNA-seq analysis of a patient treated with NP137, further support the initial view of the mode of action of the netrin-1 mAb as an inducer of tumour cell death^1,23^. Indeed, netrin-1 was previously considered to be an embryonic secreted molecule re-expressed in cancer settings to promote tumour cell survival^1,8^. NP137, by blocking netrin-1, is theorized as unleashing the pro-death activity of netrin-1 receptor as observed in various preclinical models^11–13,24^. In patients with EC or preclinical models of EC treated with NP137, this is expected to be translated into a decrease in tumour cells.

However, we have shown here that, not only does NP137 induce tumour cell death, but it also appears to impact tumour EMT. Only a few previous studies have suggested that netrin-1 may be implicated in EMT, and these showed only in vitro data^16–19^, thus only weakly supportive of a major regulatory effect of netrin-1 on EMT^25^. Tumour EMT, in which tumour cells lose their epithelial characteristics and acquire mesenchymal features, appears to be a key driver of tumour heterogeneity and has been associated with different steps in tumourigenesis such as tumour initiation, progression, metastasis and, more recently, resistance to chemotherapy or immunotherapy^2^. Whereas great progress in the understanding of the role and mechanisms by which EMT regulates these different tumour functions has been achieved, there is still virtually no pharmacological intervention that allows the clinician to alleviate EMT in tumours. It is also fair to say that, to date, because there are no clinical-stage drugs impacting only on EMT features, there is no clinical demonstration that EMT is clinically important. Here we demonstrate, using both preclinical models and pre- and on-treatment biopsies from patients with EC, that systemic treatment with NP137 led to a decrease in tumour EMT features. This inhibition of EMT features is associated with an overall more epithelial phenotype. Given the extreme complexity of tumour heterogeneity, we have not yet demonstrated whether the effect of N137 on tumour EMT is mediated by a direct effect of netrin-1 blockade on cancer cells or whether this effect is due to an indirect effect triggered by changes in the tumour microenvironment. It is likely that these effects are combined, because we have shown using single-cell analysis that changes in EMT features are associated with changes in the tumour microenvironment. Although further confirmation is required, we observed a decrease in cancer-associated fibroblasts, which usually serve as primary source of EMT-inducing molecules^26^, and a decrease in protumourigenic M2-like macrophages, which also contribute to EMT by multiple mechanisms^27^. Moreover, we observed an increase in lymphocytes endowed with cytotoxic functions and an increase in both the number and strength of interactions between immune cells and tumour cells associated with more highly efficient APCs. Together this supports the view that NP137, possibly by impacting on EMT, enhances tumour immune response. Whatever the mechanism, because there is an increasing literature describing EMT as a major player in resistance to chemotherapy and immune checkpoint inhibitors^2,28^, the observation that treatment with NP137 inhibits features of tumour EMT argues for the clinical assessment of combinations of the anti-netrin-1 mAb with conventional therapies to interfere with tumour progression. This is currently investigated in the Phase 2 GYNET trial (NCT04652076) assessing the safety and efficacy of combining NP137 with carboplatin-paclitaxel and/or pembrolizumab (anti-PD1 mAb) in patients with endometrial or cervical cancer.

## Methods

### Genetically modified mouse model and NP137 administration

Floxed homozygous *Pten* (C;129S4-Ptentm1Hwu/J, hereafter called *Pten* f/f) Cre:ER (B6.Cg-Tg(CAG-CRE/Esr1* 5Amc/J) mice were obtained from the Jackson Laboratory. Cre:ER^+/−^ *Pten* f/f mice were bred in a mixed background (C57BL6; 129S4) by crossing *Pten* f/f and Cre:ER^+/−^ mice. To obtain mice carrying both *Pten* floxed alleles (*Pten* f/f) and a single Cre:ER (Cre:ER^+/−^), Cre:ER^+/−^ *Pten* f/^+^ mice were backcrossed with *Pten* f/f mice. To induce deletion of floxed alleles, tamoxifen (Sigma-Aldrich) was dissolved in 100% ethanol at 100 mg ml^–1^. Tamoxifen solution was emulsified in corn oil (Sigma-Aldrich) at 10 mg ml^–1^ by vortexing. To induce *Pten* deletion, adult mice (4–5 weeks old) were given a single intraperitoneal injection of 0.5 mg of tamoxifen emulsion (30–35 μg mg^–1^ body weight). Three weeks after tamoxifen injection, mice were treated via intraperitoneal injection of 100 µl of NP137, or its isotypic control NP001 diluted in PBS, at 10 mg kg^–1^ every 2 days. Animal care and housing were in accordance with institutional European guidelines from the CEEA local Ethical committee of Lleida University concerning *Pten* mouse experiments. General behaviour and weight were monitored three times per week and animals were killed in the event of strong alteration or weight loss under 20%. All mice were always killed before terminal tumour progression, and endometrial tissues were analysed blind by a pathologist.

### RT–qPCR

Endometrial samples collected in the Biomedical Research institute of Lerida (Spain) were frozen and sent to the Cancer Research Center of Lyon (France) in dry ice. Samples were cryoground to obtain tumour powder, which was processed for total RNA extraction using the Nucleospin RNA Plus kit (Machery-Nagel) according to the manufacturer’s instructions. Expression of mRNA was measured using a NanoDrop1000 (Themo Scientific). RNA was retrotranscribed using the T100 ThermoCycler (Bio-Rad) and the iScript cDNA Synthesis Kit (Bio-Rad) according to the manufacturer’s instructions. RT–qPCR was performed using LC480 qPCR (Roche) and OneGreen Fast qPCR Premix (Ozyme) according to the manufacturers’ instructions.

### Bulk RNA-seq

Patient analysis: patient microbiopsy RNA was extracted with the RNA easy FFPE kit (Qiagen). The RNA-seq library was produced from 100 ng of RNA with the Illumina TruSeq Exome kit (RNA Library Prep for Enrichment & TruSeq RNA Enrichment) according to the manufacturer’s instructions and then sequenced with an Illumina NovaSeq 6000. FASTQ files were then processed with STAR (v.2.7.10a). Briefly, FASTQ files were mapped to the human reference genome (gencode.v.27) and aligned reads were converted for counting with STAR. The quality of FASTQ files was also checked, by FATSQC (v.0.11.9). RNA-seq analysis was performed with R (v.4.0.3) and the DESeq2 package (v.1.30.1). log_2_-Transformed transcripts per million were calculated, and we performed EMT score calculation as previously described^20^.

Murine endometrial cancer model: tumours were collected, snap-frozen and cryoground. RNA was extracted with a standard kit (Macherey-Nagel). The RNA-seq library was produced from RNA with Illumina TruSeq Stranded Total RNA Library Prep Human/Mouse/Rat, according to the manufacturer’s instructions, then sequenced with Illumina NOVASeq. FASTQ files were processed as described above, except for the reference genome which was Mus_musculus.GRCm38 (GENECODE release 25). Analysis was done with DESeq2 (v.1.30.1) and ggplot (v.3.1.3) packages in R (v.4.0.3). EMT scoring and heatmaps were done with log_2_ fragments per kilobase exon per million mapped reads values.

### Single-cell RNA-seq

Endometrial metastasis biopsies were dissociated for single-cell RNA-seq using the Tumor Dissociation kit by Miltenyi Biotec (no. 130–095-929). Briefly, the biopsy was placed in RPMI medium in a Petri dish on ice and cut into small pieces (2–4 mm) after removal of necrotic tissue. Pieces were then infused with the RPMI/enzyme mix (Miltenyi Biotec), transferred to a gentleMACS C tube containing RPMI/enzyme mix, attached to the sleeve of the gentleMACS Octo Dissociator and run using the programme 37_h-TDK1. After completion of the programme the cells were spun down at 300*g* for 7 min at 4 °C, resuspended in RPMI, passed through a 70 μm strainer and centrifugation was repeated. The cell pellet was treated with 500 µl of ACK solution for 5 min at room temperature and lysis then stopped with 5 ml of RPMI/10% FBS. After centrifugation, cells were resuspended in 100 µl of RPMI. The number of live cells was determined with a Luna-FL Dual fluorescence cell counter (Logos Biosystems) to obtain an expected cell recovery population of 10,000 cells per channel, loaded on a 10x G chip and run on the Chromium Controller system (10x Genomics) according to manufacturer’s instructions. Single-cell RNA-seq libraries were generated with the Chromium Single Cell 3′ v.3.1 kit (10x Genomics, no. PN-1000121) and sequenced on the NovaSeq 6000 platform (Illumina) to obtain around 50,000 reads per cell.

Except when specifically mentioned, all analyses were performed with R/Bioconductor packages, R v.4.2.2 (2022-11-10 r83330) (https://cran.r-project.org/; http://www.bioconductor.org/) in a Linux environment (x86_64-pc-linux-gnu (64-bit)).

Filtered barcoded matrices from single-cell RNA-seq data were imported into R using the Seurat package (v.4.1.1). Doublets were detected with DoubletFinder (v.2.0.3) and filtered out, together with cells showing a low number of features (nFeature_RNA < 500) or a high percentage of mitochondrial genes (above 25%). Seurat functions were used for normalization (SCTransform), merging, dimensional reduction and clustering. Initial cell type identification was based on consensus from several automated cell annotation packages (SciBet’ v.1.0, SingleR v.1.10.0 and scType (https://github.com/IanevskiAleksandr/sc-type/blob/master/README.md)). T cell subtypes were visually inspected and manually curated after further annotation with ProjecTILs (v.3.0.3) and the python implementation of CellTypist (v.1.3.0). EMT signature scores were calculated by three methods (Seurat’s AddModuleScore function, UCell (v.2.2.0) and AUCell (v.1.18.1)) and using two different gene lists (MsigDb Hallmark EMT pathway and the PanCancer EMT signature^20^). Pathway enrichment analyses were performed with the ‘escape’ package (v.1.6.0). Additional visualizations were based on functions from Nebulosa (v.1.6.0), Scillus (v.0.5.0) and ggplot2 (v.3.3.6).

Spatial RNA-seq matrices and images were imported into R using the Load10X_Spatial function of the Seurat package. For each sample, only those spots with more than 1,000 features were kept for downstream preprocessing, including SCTransform normalization, dimensional reduction and clustering. Most analyses were performed independently for each sample (without merging or integration). Two strategies were used for cell type annotation: label transfer following integration of the single-cell RNA-seq data described above, and manual annotation using known markers for major cell types.

Inference of cell–cell communication was done with CellChat (v.1.6.0), for both single-cell and spatial RNA-seq data.

### Histology and IHC analysis

Cre:ER^+/−^ *Pten* f/f mice were euthanized by cervical dislocation after 3 weeks of NP137 treatment. Endometrial samples were collected and formalin fixed overnight at 4 °C. Tumours were paraffin embedded for further histologic analysis. Paraffin blocks were sectioned at 3 µm and dried for 1 h at 65 °C before the pretreatment procedures of deparaffinization, rehydration and epitope retrieval in the pretreatment module at 95 °C for 20 min in 50× Tris/EDTA buffer. Before staining of sections, endogenous peroxidase was blocked. Representative images were taken with a Leica DMD108 microscope.

Immunohistochemistry was performed on an automated immunostainer (Ventana discoveryXT, Roche) using the rabbit Omni map DAB Kit. Sections were incubated with specific antibodies targeting EpCAM (no. ab71916, abcam), cleaved caspase-3 (no. 9661, Cell Signaling Technologies), netrin-1 (no. CPA2389, Cohesion Biosciences), Unc5B (no. 13851S, Cell Signaling Technologies) and CD8 (no. 4SM15, eBioscience). Staining was by anti-rabbit horseradish peroxidase, visualized with 3,3′-diaminobenzidine as a chromogenic substrate and counterstained with Gill’s haematoxylin. Histological quantifications were performed with Halo software (Indica Labs).

### Multiplex IHC

Sequential chromogenic multiplex IHC for vimentin/pancytokeratin (panCK), as previously described, was performed on tumour sections from patients included in the NP137 clinical trial and that were collected at C1D1 and C3D1. Dewaxed 4-μm-thick, paraffin-embedded tissue sections were subjected to two successive steps of IHC on a Ventana discovery XT platform (Ventana, Roche Diagnostics) using the REDMap and DABMap detection systems according to the manufacturer’s recommendations. In a first step for vimentin expression, slides were incubated with mouse monoclonal anti-human vimentin for 1 h (mouse, clone V9, Leica, no. NCL-L-VIM-V9, dilution 1:100) and incubated with rabbit monoclonal anti-mouse secondary antibody for 20 min (clone M1gG51-4, abcam, no. 125913, dilution 1:750). The slides were then incubated with biotinylated anti-rabbit secondary antibody for 24 min (Vector Laboratories, dilution 1:200) followed by the addition of the streptavidin–alkaline phosphatase complex. Immunostaining was detected by incubation with naphthol and Fast red. Tissue sections were counterstained with Gill’s haematoxylin, dehydrated and mounted. Whole histological slides were digitized at ×20 magnification using a Hamamatsu 2.0 HT scanner. After removal of coverslips, slides were incubated in 100% ethanol until complete erasure of red colour. In a second step, to show panCK expression, the slides were incubated with mouse monoclonal anti-panCK antibody for 1 h (mouse, clone CKAE1AE3, Dako Belgium, no. M351529-2, dilution 1:150) and then with rabbit monoclonal anti-mouse secondary antibody for 20 min. The slides were then incubated with biotinylated anti-rabbit secondary antibody for 28 min (Vector Laboratories, dilution 1:200) followed by the addition of streptavidin–alkaline phosphatase. PanCK immunostaining was detected by incubation with naphthol and Fast red. The IHC slides were counterstained with Gill’s haematoxylin, dehydrated, mounted and again digitized. Image processing and analysis for Hynrid EMT score computation were performed using Visiomorph DP 2018.4 to determine vimentin and panCK co-expression in each tissue slide. Briefly, each pair of vimentin- and panCK-labelled virtual slides, which were acquired from the same tissue section, was subjected to image registration. Vimentin- and panCK-positive areas were automatically detected in the aligned virtual slides to evidence their co-expression in tumour cells. This co-expression was evaluated on whole slides at ×10 magnification to take into account potential imperfections. Manual corrections were carried out to exclude irrelevant sample parts, such as necrosis. Cell nuclei negative for both markers were also excluded, to focus only on cytoplasmic areas where colocalization could occur.

### Spatial transcriptomics using Visium FFPE technology

FFPE tissue sections were placed on Visium slides and prepared according to the 10x Genomics protocols. After H&E staining, imaging and de-crosslinking steps, tissue sections were incubated with human-specific probes targeting 17,943 genes (10x Genomics, Visium Human Transcriptome Probe Set v.1.0). Probes hybridized on mRNA were captured on Visium slides and a gene xpression library prepared following the provided protocol and sequenced on an Illumina NovaSeq 6000 with 50,000 reads per spot targeted sequencing depth.

For each FFPE section, FASTQ files and histology images were processed using 10x Space Ranger v.2.0 to obtain the gene expression matrix associated with each spot.

Seurat v.4 (https://satijalab.org/seurat/) in R 4.1 was used to perform the analysis. Briefly, filtered matrices were loaded, merged per patient and spots with fewer than 1,000 detected genes were removed. Following SCTransform normalization we subset the tumoural spot according to pathologist spot identification then calculated the EMT gene set enrichment score (escape R package) with the UCell method.

### NP137 clinical trial

NP137 is a first-in-human Phase I trial with a dose-escalation part followed by extension cohorts (NCT02977195) conducted in adult patients with advanced or metastatic solid tumours. The dose-escalation part was initiated using an accelerated dose titration with one patient per dose level until the occurrence of a grade 2 or higher drug-related adverse event. Following occurrence of a grade 2 NP137-related adverse event, patients were enrolled in a slower dose-escalation design with at least three patients per dose level using a modified continual reassessment method. Additional patients were enrolled in three biomarker cohorts, at dose levels that had been declared safe, and underwent paired biopsies for pharmacodynamics purposes. In the dose-escalation part, 19 patients were enrolled in seven dose levels (1–20 mg kg^–1^, intravenous, Q2W). No dose-limiting toxicities were observed and 11 (58%) patients had infusion-related reactions of grade 12 severity, all at doses of 4 mg kg^–1^ and above^14^. Based on available data, 14 mg kg^–1^ Q2W was selected as the recommended Phase 2 dose. Two extension cohorts were opened, including one in patients with hormone receptor-positive EC (enrolment closed in October 2021).

The trial was conducted according to Good Clinical Practice guidelines, the Declaration of Helsinki and relevant French and European laws and directives. All patients provided written informed consent.

### Statistical analysis

Statistical analyses were performed on Prism (GraphPad Software). In the figure legends, *n* denotes the total number of replicates. All statistical tests were two-sided. For mouse experiments, statistical methods were not used to predetermine necessary sample size but sample size was chosen based on pilot experiments applying appropriate statistical tests that could return significant results. Survival curves were analysed using the log-rank (Mantel–Cox) test. Population ratios were analysed by chi-squared tests. qPCR expression, caspase-3 IHC and thyroid weights were compared by Mann–Whitney test. For data involving patients, gene expressions and EMT scores were compared by *t*-test.

### Reporting summary

Further information on research design is available in the Nature Portfolio Reporting Summary linked to this article.

## Online content

Any methods, additional references, Nature Portfolio reporting summaries, source data, extended data, supplementary information, acknowledgements, peer review information; details of author contributions and competing interests; and statements of data and code availability are available at 10.1038/s41586-023-06367-z.

### Supplementary information

Supplementary Table 1 Clinical information for patients analysed in the scope of this study. Three tables describe the clinical data of all patients analysed for NTN1/UNC5 expression (Supplementary Table 1a), EC treated in the Phase 1 clinical trial (Supplementary Table 1b) and the baseline characteristics of these patients (Supplementary Table 1c).

Reporting Summary

### Source data

Source Data Fig. 1

Source Data Fig. 2

Source Data Fig. 3

Source Data Fig. 4

Source Data Extended Data Fig. 1

Source Data Extended Data Fig. 2

Source Data Extended Data Fig. 3

Source Data Extended Data Fig. 5

Source Data Extended Data Fig. 7

## Figures and Tables

**Extended Data Table 1:**
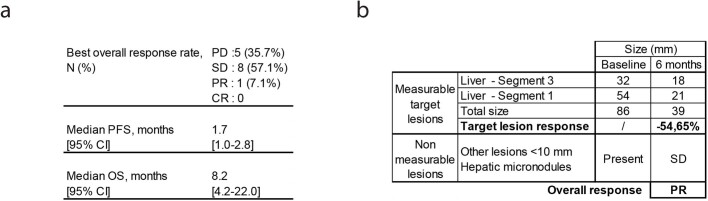
Clinical data for 14 patients with endometrium carcinoma enrolled in NP137 trial

**a**, Median progression-free survival (mPFS) and overall survival (mOS) are presented with their 95% confidence interval (95% CI). Best overall response is also presented. N: number of patients. **b**, Table resuming target lesion size evolution and non-target lesions from baseline to 6 months of patient no. 02.004. PR indicates partial response, SD indicates stable disease as per RECIST V1.1. PR was identified both by investigator review and by centralized review.

**Extended Data Table 2:**
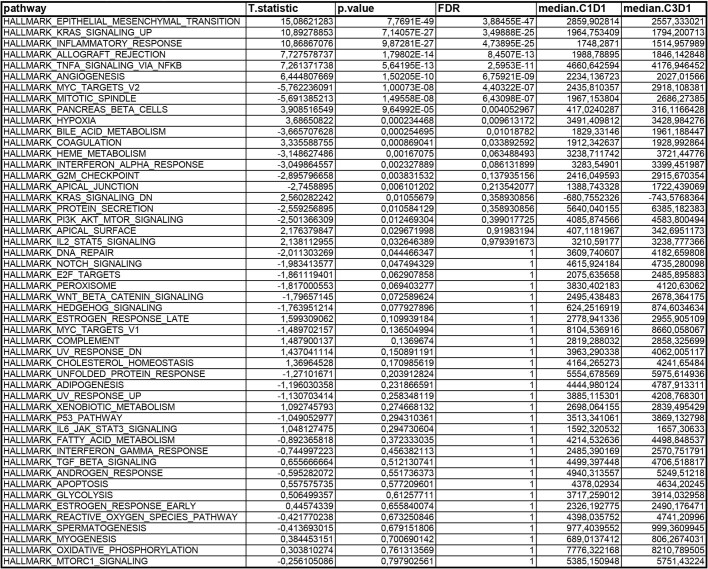
Hallmark pathway analysis extracted from scRNAseq from patient 01-040

Table presenting a gene set enrichment between C3D1 and C1D1 tumoral cluster cell for all the hallmark gene sets. – Note that HALLMARK_EPITHELIAL_MESENCHYMAL_TRANSITION is the strongest enriched pathway (two-sided student test).

## Data Availability

The raw RNA-seq, single-cell RNA-seq and spatial transcriptomic sequencing data of this study have been deposited in Gene Expression Omnibus with accession no. GSE225691. For version control, sharing and reproducibility, all bioinformatic code related to the single-cell analysis is retained in a GitHub repository (https://github.com/hernandezvargash/NP137_single.cell). Source data are provided with this paper.
