# Supplementary material for: Netrin-1 blockade inhibits tumour growth and EMT features in endometrial cancer
Source: Nature. 2023 Aug 2;620(7973):409–16. doi: 10.1038/s41586-023-06367-z (PMC10412451; doi:10.1038/s41586-023-06367-z)
Supplement: Supplementary file 1 — Clinical information for patients analysed in the scope of this study. Three tables describe the clinical data of all patients analysed for NTN1/UNC5 expression (Supplementary Table 1a), EC treated in the Phase 1 clinical trial (Supplementary Table 1b) and the baseline characteristics of these patients (Supplementary Table 1c). [file 41586_2023_6367_MOESM1_ESM.pdf]

nature portfolio

https://doi.org/10.1038/s41586-023-06367-z

Supplementary information

Netrin-1 blockade inhibits tumour growth and EMT features in endometrial cancer

In the format provided by the authors and unedited

nature portfolio

https://doi.org/10.1038/s41586-023-06367-z

Supplementary information

Netrin-1 blockade inhibits tumour growth and EMT features in endometrial cancer

In the format provided by the authors and unedited

Table 1a - Clinical data from EC retrospectivce cohort analysis

Patient ID	T/N	Age at diagnosis	Histological type	Grade	Stage
ENDO-04	Tumoral	84	Endometrioid	1	Ib
ENDO-07	Tumoral	50	Endometrioid	1	Ia
ENDO-12	Tumoral	56	Endometrioid	1	Ia
ENDO-16	Tumoral	82	Endometrioid	1	Ia (T1aN0)
ENDO-18	Tumoral	67	Endometrioid	1	Ia (T1aN0)
ENDO-24	Tumoral	63	Endometrioid	1	Ia (T1aN0)
ENDO-25	Tumoral	61	Endometrioid	1	Ia (T1aN0)
ENDO-26	Tumoral	35	Endometrioid	1	Ia (T1aN0)
ENDO-30	Tumoral	62	Endometrioid	1	Ib (T1bN0)
ENDO-35	Tumoral	79	Endometrioid	1	Ia (T1aNx)
ENDO-36	Tumoral	79	Endometrioid	1	Ib (T1bN0)
ENDO-38	Tumoral	43	Endometrioid	1	Ia (T1aNx)
ENDO-40	Tumoral	63	Endometrioid	1	Ib (T1bN0)
ENDO-41	Tumoral	80	Endometrioid	1	Ib (T1bN0)
ENDO-48	Tumoral	66	Endometrioid	1	Ia (T1aN0)
ENDO-63	Tumoral	68	Endometrioid	1	T3a N0
ENDO-64	Tumoral	64	Endometrioid	1	T1a Nx
ENDO-65	Tumoral	56	Endometrioid	1	T1a N0
ENDO-06	Tumoral	72	Endometrioid	2	II (T2N0)
ENDO-08	Tumoral	66	Endometrioid	2	Ib
ENDO-09	Tumoral	58	Endometrioid	2	Ia
ENDO-14	Tumoral	57	Endometrioid	2	Ib
ENDO-15	Tumoral	71	Endometrioid	2	Ib (T1bN0)
ENDO-17	Tumoral	71	Endometrioid	2	Ia (T1aNx)
ENDO-22	Tumoral	61	Endometrioid	2	IIIc (T1bN1)
ENDO-23	Tumoral	71	Endometrioid	2	Ib (T1bN0)
ENDO-27	Tumoral	61	Endometrioid	2	Ia (T1aN0)
ENDO-29	Tumoral	74	Endometrioid	2	IIIc (T1bN1)
ENDO-34	Tumoral	71	Endometrioid	2	IIIc (T2N1)
ENDO-39	Tumoral	84	Endometrioid	2	Ib (T1bNx)
ENDO-43	Tumoral	86	Endometrioid	2	Ia (T1aNx)
ENDO-46	Tumoral	69	Endometrioid	2	Ib (T1bNx)
ENDO-49	Tumoral	72	Endometrioid	2	Ia(T1aNx)
ENDO-50	Tumoral	73	Endometrioid	2	Ib (T1bN0)
ENDO-51	Tumoral	52	Endometrioid	2	Ia (T1aN0)
ENDO-58	Tumoral	71	Endometrioid	2	IIIc (T2N1)
ENDO-60	Tumoral	52	Endometrioid	2	IIIa (T3aN0)
ENDO-67	Tumoral	68	Endometrioid	2	T1a N0
ENDO-56	Tumoral	75	Carcinosarcoma	3	Ia (T1aNx)
ENDO-03	Tumoral	80	CCC	3	IIIc (T3aN1)
ENDO-05	Tumoral	71	Endometrioid	3	Ib
ENDO-11	Tumoral	60	Endometrioid	3	Ia
ENDO-13	Tumoral	59	Endometrioid	3	IIIc (T1bN1)
ENDO-32	Tumoral	81	Endometrioid	3	Ib (T1bNx)
ENDO-33	Tumoral	66	Endometrioid	3	Ib (T1bN0)
ENDO-44	Tumoral	88	Endometrioid	3	II (T2Nx)
ENDO-45	Tumoral	60	Endometrioid	3	IB (T1bN0)
ENDO-59	Tumoral	91	Endometrioid	3	IIIc (T3bN1)
ENDO-66	Tumoral	55	Endometrioid	3	T1b N0
ENDO-69	Tumoral	73	Endometrioid	3	T1a N0
ENDO-70	Tumoral	89	Endometrioid	3	T1a Nx
ENDO-71	Tumoral	47	Endometrioid	3	T2N0
ENDO-72	Tumoral	68	Endometrioid	3	T1a N0
ENDO-01	Tumoral	72	UPSC	3	IIIc (T2aN1)
ENDO-02	Tumoral	72	UPSC	3	IIIa (T3aN0)
ENDO-31	Tumoral	81	UPSC	3	Ia (T1aNx)
ENDO-37	Tumoral	81	UPSC	3	T1bNx
ENDO-47	Tumoral	82	UPSC	3	IIIa (T3aNx)
ENDO-57	Tumoral	68	UPSC	3	IIIc (T2N2)
ENDO-62	Tumoral	75	UPSC	3	1a (T1aN0)
ENDO-68	Tumoral	62	UPSC	3	T1a N0
ENDO-10	Tumoral	53	Endometrioide + UPSC	1 +3	IIIc (T1aN1)
ENDO-42	Tumoral	74	Endometrioide + UPSC	2+3	Ib (T1bN0)
ENDO-28	Tumoral	69	Endometrioide + UPSC	3+3	Ivb (T3aNxM1)
ENDO-76	Normal	65		atrophia	
ENDO-75	Normal	53		hypotrophia	
ENDO-73	Normal	47		proliferative	
ENDO-74	Normal	43		proliferative	
ENDO-77	Normal	44		proliferative	
ENDO-78	Normal	50		proliferative	
ENDO-79	Normal	41		proliferative	
ENDO-80	Normal	45		proliferative	

Table 1b - Clinical data and translationnal analysis from 14 patients with EC treated in the Phase I clinical trial

Patient ID	Dose of NP137 (mg/Kg)	Age at the Inclusion	Histologic Type	Grade at initial diagnosis	Stade (AJCC)	Bulk RNAseq	IHC EpCAM	Multiplex IHC PanCK/Vim	scRNAseq/ 10x Visium
1-024	14	44	Endometrioid ADK	2	IVB	x			
1-028	20	73	Papillary serous ADK	3	IVB	x	x		
1-030	14	69	Endometrioid ADK	2	IVB	x	x	x	
1-032	14	79	Endometrioid ADK	2	IVB	x			
1-034	14	48	Endometrioid ADK	2	NA	x	x	x	x
1-035	14	66	Serous ADK	3	NA		x		
1-039	14	80	Papillary serous ADK	3	NA	x	x	x	x
1-040	14	70	Endometrioid ADK	2	NA	x	x	x	x
2-004	14	74	Endometrioid ADK	2	IV	No biopsy			
2-012	20	79	Endometrioid ADK	1	IV	x			
2-013	20	66	Papillary serous ADK	3	IV	x			
2-014	14	65	Endometrioid ADK	2	IV	x			
2-015	14	65	Endometrioid ADK	2	IVB	x			
2-020	14	61	Poorly differentiated ADK	3	NA	x			

Table 1c - Baseline characteristics from 14 patients with EC treated in the Phase I clinical trial

**Age at inclusion**	
Median, years [min-max]	68.3 [44.7-80.6]

**PS ECOG at inclusion,** n (%)	
0	5 (35.7%)
1	9 (64.3%)

**AJCC Stage, n (%)**	
IV	14 (100%)

**Surgery of primary tumor**	13 (92.3%)

**Radiotherapy of primary tumor**	10 (71.4%)

**Prior systemic treatment**	
Median [min-max]	3.0 [2.0 - 6.0]
2-3L, n (%)	9 (64.3%)
>3L, n (%)	5 (35.7%)

**Metastatic site at inclusion**	
Median [min-max]	2.0 [1.0 - 5.0]

**Main metastatic sites, N (%)**	
Lymph node	8 (57.1%)
Lung	8 (57.1%)
Liver	6 (42.9%)

Supplementary Table 1.

Table presenting clinical characteristics of patients analyzed in the scoop of this manuscript. **a**, details on patients of the cohort of 72 human endometrial tumors used to monitor netrin-1/UNC5B expression**. b**, Patients with endometrial adenocarcinoma included in the NP137 trial from whom we described clinical data and from whom biopsies were collected for either bulk RNAseq, EpCAM IHC, multiplex IHC PanCytokeratin and Vimentin or scRNAseq/Visium. Due to material availability, not all the technics could be performed for all patients. **c**, Patient's baseline clinical characteristics before inclusion in the NP137 trial.
