# Supplementary material for: Netrin-1 blockade inhibits tumour growth and EMT features in endometrial cancer
Source: Nature. 2023 Aug 2;620(7973):409–16. doi: 10.1038/s41586-023-06367-z (PMC10412451; doi:10.1038/s41586-023-06367-z)
Supplement: Supplementary file 2 — Reporting Summary [file 41586_2023_6367_MOESM2_ESM.pdf]

nature portfolio

Corresponding author(s): DBPR 2022-04-06277B

Last updated by author(s): May 24, 2023

Reporting Summary

Nature Portfolio wishes to improve the reproducibility of the work that we publish. This form provides structure for consistency and transparency in reporting. For further information on Nature Portfolio policies, see our Editorial Policies and the Editorial Policy Checklist.

Statistics

For all statistical analyses, confirm that the following items are present in the figure legend, table legend, main text, or Methods section.

n/a	Confirmed	
☐	☒	The exact sample size (n) for each experimental group/condition, given as a discrete number and unit of measurement
☐	☒	A statement on whether measurements were taken from distinct samples or whether the same sample was measured repeatedly
☐	☒	The statistical test(s) used AND whether they are one- or two-sided<br>*Only common tests should be described solely by name; describe more complex techniques in the Methods section.*
☒	☐	A description of all covariates tested
☒	☐	A description of any assumptions or corrections, such as tests of normality and adjustment for multiple comparisons
☐	☒	A full description of the statistical parameters including central tendency (e.g. means) or other basic estimates (e.g. regression coefficient) AND variation (e.g. standard deviation) or associated estimates of uncertainty (e.g. confidence intervals)
☒	☐	For null hypothesis testing, the test statistic (e.g. F, t, r) with confidence intervals, effect sizes, degrees of freedom and P value noted<br>*Give P values as exact values whenever suitable.*
☒	☐	For Bayesian analysis, information on the choice of priors and Markov chain Monte Carlo settings
☒	☐	For hierarchical and complex designs, identification of the appropriate level for tests and full reporting of outcomes
☒	☐	Estimates of effect sizes (e.g. Cohen's d, Pearson's r), indicating how they were calculated

Our web collection on statistics for biologists contains articles on many of the points above.

Software and code

Policy information about availability of computer code

Data collection	NA
Data analysis	For the RNAseq, we used a classical pipeline, alignment and mapping with the STAR software (v2.5.2b), the analysis was performed with the DeSEQ2 package (ver 1.30.1) with the R software (ver4.0.3).<br>Concerning the single cell RNAseq analysis Spatial transcrriptomic, we used a classical pipeline, CellRanger (ver6.1.1) or Space Ranger v2.0 to generate the counting matrices and performed the analysis with Seurat(ver4.1.1) on R sofware (Ver4.2.0). During the analysis, we used DoubletFinder (ver2.0.3), severals annotations packages (SciBet v.1.0, SingleR v.1.10.0, scType, ProjecTILs ver.3.0.3, CellTypist v.1.3.0), UCell ver2.2.0, AUCell v.1.18.1, escape package v.1.6.0, Nebulosa ver.1.6.0, Scillus'ver0.5.0, ggplot2 v.3.3.6. For cell communication , we used cellchat v.1.6.0.

For manuscripts utilizing custom algorithms or software that are central to the research but not yet described in published literature, software must be made available to editors and reviewers. We strongly encourage code deposition in a community repository (e.g. GitHub). See the Nature Portfolio guidelines for submitting code & software for further information.

Data

Policy information about availability of data

All manuscripts must include a data availability statement. This statement should provide the following information, where applicable:

- Accession codes, unique identifiers, or web links for publicly available datasets
- A description of any restrictions on data availability
- For clinical datasets or third party data, please ensure that the statement adheres to our policy

We are currently in the process of depositing the data on the GEOdataset. We do not have an accession number yet.

Field-specific reporting

Please select the one below that is the best fit for your research. If you are not sure, read the appropriate sections before making your selection.

☒ Life sciences ☐ Behavioural & social sciences ☐ Ecological, evolutionary & environmental sciences

For a reference copy of the document with all sections, see nature.com/documents/nr-reporting-summary-flat.pdf

Life sciences study design

All studies must disclose on these points even when the disclosure is negative.

Sample size	In the dose escalation part: it was planned to enroll a maximum of 30 patients in 7 dose level (DL) cohorts and a maximum of 24 patients in biological cohort starting at DL4. In each extension cohorts, it was planned to enroll 14 patients.<br>No sample size calculation were performed in the preclinical part of the study. Sample sizes were determined according to the standards of publication in the respective models of prclinical research. Concerening clinical data, sample size directly belonged to patient inclusions and sample availability.
Data exclusions	No data exclusion had to be done.
Replication	No troubles in experiment replication have to be declared. All the replicates are included in the manuscript.
Randomization	Randomization was performed for all animal experiments. In PTEN mouse experiments, mice were randomly attributed to the differents groups of treatment without specific criterias. In xenograft experiments, around 100mm3 of tumor volume, mice were allocated to the different groups by minimizing the difference in mean volume and SEM.
Blinding	Pathological samples analysis were conducted in blind for tumor complexity assesment or cleaved caspase-3 IHC quantifications.

Reporting for specific materials, systems and methods

We require information from authors about some types of materials, experimental systems and methods used in many studies. Here, indicate whether each material, system or method listed is relevant to your study. If you are not sure if a list item applies to your research, read the appropriate section before selecting a response.

Materials & experimental systems

n/a	Involved in the study	
☐	☒	Antibodies
☐	☒	Eukaryotic cell lines
☐	☐	Palaeontology and archaeology
☐	☒	Animals and other organisms
☐	☒	Human research participants
☐	☒	Clinical data
☐	☐	Dual use research of concern

Methods

n/a	Involved in the study	
☐	☐	ChIP-seq
☐	☐	Flow cytometry
☐	☐	MRI-based neuroimaging

Antibodies

Antibodies used	anti-Unc5B, used for IHC, clone D9M72, Cell Signaling Technologies, ref.#13851S, batch 1.<br>anti-Ntn1, used for IHC, Cohesion Biosciences, ref.#CPA2389.<br>anti-Cleaved Caspase 3, used for IHC, Cell Signaling Technologies, ref.#9661, batch 45.<br>anti-EpCam, used for IHC, Abcam, ref.#ab71916, batch GR3266477-1.
Validation	Every antibodies used are validated for species and applications on manufacturer's website.

Eukaryotic cell lines

Policy information about cell lines

Cell line source(s)	Ishikawa cell line is from ECACC, authentification 99040201.<br>ARK1 and ARK2 cell lines were obtained from Alessendro Satin (Yale University), authetification USPC-ARK1 and USPC-ARK2.
Authentication	Non of the cell lines have been authentificated after being purchased or acquired.
Mycoplasma contamination	Cell lines are regularly tested for mycoplasma contamination. No contaminations were observed over the experimental period.

Commonly misidentified lines (See ICLAC register)	To our knowledge, no commonly misidentified cell lines were used in this study.

Palaeontology and Archaeology

Specimen provenance	*Provide provenance information for specimens and describe permits that were obtained for the work (including the name of the issuing authority, the date of issue, and any identifying information). Permits should encompass collection and, where applicable, export.*
Specimen deposition	*Indicate where the specimens have been deposited to permit free access by other researchers.*
Dating methods	*If new dates are provided, describe how they were obtained (e.g. collection, storage, sample pretreatment and measurement), where they were obtained (i.e. lab name), the calibration program and the protocol for quality assurance OR state that no new dates are provided.*

☐ Tick this box to confirm that the raw and calibrated dates are available in the paper or in Supplementary Information.

Ethics oversight	*Identify the organization(s) that approved or provided guidance on the study protocol, OR state that no ethical approval or guidance was required and explain why not.*

Note that full information on the approval of the study protocol must also be provided in the manuscript.

Animals and other organisms

Policy information about studies involving animals; ARRIVE guidelines recommended for reporting animal research

Laboratory animals	For PTEN experiments, mice were C57b6 injected with tamoxifen at weeks old and maintained in facility for 6-7 additionnal weeks before being sacrificied. For xenograft experiments, 7 weeks old BalbC Nude mice were injected with Ishikawa cell line and sacrificied 1 month later.
Wild animals	The study did not involve wild animals.
Field-collected samples	No fied-collected samples were used in this paper.
Ethics oversight	Animal care and housing were in accordance with institutional European guidelines as put forth by the CECCAP local Ethical Committee (CLB_2014_012) for xenograft experiments and the CEEA local Ethical committee of Lleida University concerning PTEN mouse experiments.

Note that full information on the approval of the study protocol must also be provided in the manuscript.

Human research participants

Policy information about studies involving human research participants

Population characteristics	Clinical trial : Main eligibility criteria were : adult patients suffering from histologically confirmed locally advanced / metastatic: i) Dose escalation cohorts and biological cohorts : solid tumors of any histological types, ii) extension cohort 1: endometrium or cervix carcinoma and iii) extension 2: endometrial carcinoma and who positively expressed Hormone Receptors (with a positivity threshold value ≥ 10%); with documented disease progression after at least one prior line of treatment in the metastatic/advanced setting; RECIST V1.1 evaluable disease, for extension cohorts: at least one lesion with a diameter ≥10 mm, visible by medical imaging and accessible to percutaneous sampling, signed inform consent, willingness to use adequate contraception method.
Recruitment	The recruitement is closed. In the dose escalation part : 42 patients were enrolled (n=19 patients were enrolled in 7 dose level cohorts + 23 in biological collection cohorts). In each extension cohort : 14 patients were enrolled.<br>Patients were enrolled in 3 comprehensive cancer center in France (Centre Léon, Bérard, IUCT-Oncopole Toulouse and Insittut de Canérologie de l'Ouest).
Ethics oversight	The protocol, its amendment and Inform consent form were approved by an Ethic commiteee (Comité de Protection des Personnes SUD-EST IV). The clinical trial NP137 was conducted in accordance with the principles laid down by the 18th World Clinical trial : Medical Assembly (Helsinki, 1964) and all applicable amendments laid down by the World Medical Assemblies and the ICH guidelines E6 (R2) for Good Clinical Practice (GCP). This Clinical Trial was onducted in compliance with the French and European laws and regulations in force, including GPDR.<br><br>For the cohort of 72 human endometrial tumors : All experiments were performed in accordance with relevant guidelines and regulations.Research involving these human samples must have been performed in accordance with the WMA Declaration of Helsinki.

Note that full information on the approval of the study protocol must also be provided in the manuscript.

Clinical data

Policy information about clinical studies

All manuscripts should comply with the ICMJE guidelines for publication of clinical research and a completed CONSORT checklist must be included with all submissions.

Clinical trial registration	ClinicalTrials.gov Identifier:NCT02977195
Study protocol	A summary of the protocol is available at https://clinicaltrials.gov/ct2/show/NCT02977195?term=NP137&draw=2&rank=1
Data collection	Clinical data were collected into dedicated eCRF (ENNOV CLinical) and fully monitored on site for all enrolled patients. Database lock for dose escalation and biological cohorts was done on 16/07/2019 and for extension 01 on 25 March 2020. Extension 02 is not yet analysed (database lock planned by June 2021)
Outcomes	PRIMARY OUTCOME<br>- Dose escalation part : Dose limiting toxicities over the first 28d of treatment.<br>- Extension Part: Objective response rate after 3 months (ORR3m) defined as the rate of patients with CR or PR as per RECIST 1.1 after 3 months of treatment.<br>SECONDARY OUTCOMES: Safety graded according to NCI-CTCAE, Version 4.03; Overall response Rate (ORR) according to RECIST V1.1; duration of response, progression-free survival; NP137 pharmacokinetic parameters (Cmax ; tmax ; AUCt ; AUC∞ and t½); PFS2/PFS1 (for expansion part only) defined as the ratio of the PFS of the current treatment (PFS2) versus the PFS of previous treatment before inclusion (PFS1); Tumor Growth Kinetics by comparing post-treatment scans with at least 2 pre-treatment scans.

Dual use research of concern

Policy information about dual use research of concern

Hazards

Could the accidental, deliberate or reckless misuse of agents or technologies generated in the work, or the application of information presented in the manuscript, pose a threat to:

No	Yes	
☒	☐	Public health
☒	☐	National security
☒	☐	Crops and/or livestock
☒	☐	Ecosystems
☒	☐	Any other significant area

Experiments of concern

Does the work involve any of these experiments of concern:

No	Yes	
☒	☐	Demonstrate how to render a vaccine ineffective
☒	☐	Confer resistance to therapeutically useful antibiotics or antiviral agents
☒	☐	Enhance the virulence of a pathogen or render a nonpathogen virulent
☒	☐	Increase transmissibility of a pathogen
☒	☐	Alter the host range of a pathogen
☒	☐	Enable evasion of diagnostic/detection modalities
☒	☐	Enable the weaponization of a biological agent or toxin
☒	☐	Any other potentially harmful combination of experiments and agents

ChIP-seq

Data deposition

☐ Confirm that both raw and final processed data have been deposited in a public database such as GEO.

☐ Confirm that you have deposited or provided access to graph files (e.g. BED files) for the called peaks.

Data access links<br>*May remain private before publication.*	*For "Initial submission" or "Revised version" documents, provide reviewer access links. For your "Final submission" document, provide a link to the deposited data.*
Files in database submission	*Provide a list of all files available in the database submission.*

Genome browser session
(e.g. UCSC)

Provide a link to an anonymized genome browser session for "Initial submission" and "Revised version" documents only, to enable peer review. Write "no longer applicable" for "Final submission" documents.

Methodology

Replicates

Describe the experimental replicates, specifying number, type and replicate agreement.

Sequencing depth

Describe the sequencing depth for each experiment, providing the total number of reads, uniquely mapped reads, length of reads and whether they were paired- or single-end.

Antibodies

Describe the antibodies used for the ChIP-seq experiments; as applicable, provide supplier name, catalog number, clone name, and lot number.

Peak calling parameters

Specify the command line program and parameters used for read mapping and peak calling, including the ChIP, control and index files used.

Data quality

Describe the methods used to ensure data quality in full detail, including how many peaks are at FDR 5% and above 5-fold enrichment.

Software

Describe the software used to collect and analyze the ChIP-seq data. For custom code that has been deposited into a community repository, provide accession details.

Flow Cytometry

Plots

Confirm that:

☐ The axis labels state the marker and fluorochrome used (e.g. CD4-FITC).

☐ The axis scales are clearly visible. Include numbers along axes only for bottom left plot of group (a 'group' is an analysis of identical markers).

☐ All plots are contour plots with outliers or pseudocolor plots.

☐ A numerical value for number of cells or percentage (with statistics) is provided.

Methodology

Sample preparation

Describe the sample preparation, detailing the biological source of the cells and any tissue processing steps used.

Instrument

Identify the instrument used for data collection, specifying make and model number.

Software

Describe the software used to collect and analyze the flow cytometry data. For custom code that has been deposited into a community repository, provide accession details.

Cell population abundance

Describe the abundance of the relevant cell populations within post-sort fractions, providing details on the purity of the samples and how it was determined.

Gating strategy

Describe the gating strategy used for all relevant experiments, specifying the preliminary FSC/SSC gates of the starting cell population, indicating where boundaries between "positive" and "negative" staining cell populations are defined.

☐ Tick this box to confirm that a figure exemplifying the gating strategy is provided in the Supplementary Information.

Magnetic resonance imaging

Experimental design

Design type

Indicate task or resting state; event-related or block design.

Design specifications

Specify the number of blocks, trials or experimental units per session and/or subject, and specify the length of each trial or block (if trials are blocked) and interval between trials.

Behavioral performance measures

State number and/or type of variables recorded (e.g. correct button press, response time) and what statistics were used to establish that the subjects were performing the task as expected (e.g. mean, range, and/or standard deviation across subjects).

Acquisition

Imaging type(s)	*Specify: functional, structural, diffusion, perfusion.*
Field strength	*Specify in Tesla*
Sequence & imaging parameters	*Specify the pulse sequence type (gradient echo, spin echo, etc.), imaging type (EPI, spiral, etc.), field of view, matrix size, slice thickness, orientation and TE/TR/flip angle.*
Area of acquisition	*State whether a whole brain scan was used OR define the area of acquisition, describing how the region was determined.*

Diffusion MRI ☐ Used ☐ Not used

Preprocessing

Preprocessing software	*Provide detail on software version and revision number and on specific parameters (model/functions, brain extraction, segmentation, smoothing kernel size, etc.).*
Normalization	*If data were normalized/standardized, describe the approach(es): specify linear or non-linear and define image types used for transformation OR indicate that data were not normalized and explain rationale for lack of normalization.*
Normalization template	*Describe the template used for normalization/transformation, specifying subject space or group standardized space (e.g. original Talairach, MNI305, ICBM152) OR indicate that the data were not normalized.*
Noise and artifact removal	*Describe your procedure(s) for artifact and structured noise removal, specifying motion parameters, tissue signals and physiological signals (heart rate, respiration).*
Volume censoring	*Define your software and/or method and criteria for volume censoring, and state the extent of such censoring.*

Statistical modeling & inference

Model type and settings	*Specify type (mass univariate, multivariate, RSA, predictive, etc.) and describe essential details of the model at the first and second levels (e.g. fixed, random or mixed effects; drift or auto-correlation).*
Effect(s) tested	*Define precise effect in terms of the task or stimulus conditions instead of psychological concepts and indicate whether ANOVA or factorial designs were used.*

Specify type of analysis: ☐ Whole brain ☐ ROI-based ☐ Both

Statistic type for inference (See Eklund et al. 2016)	*Specify voxel-wise or cluster-wise and report all relevant parameters for cluster-wise methods.*
Correction	*Describe the type of correction and how it is obtained for multiple comparisons (e.g. FWE, FDR, permutation or Monte Carlo).*

Models & analysis

n/a	Involved in the study
☐	☐ Functional and/or effective connectivity
☐	☐ Graph analysis
☐	☐ Multivariate modeling or predictive analysis

Functional and/or effective connectivity	*Report the measures of dependence used and the model details (e.g. Pearson correlation, partial correlation, mutual information).*
Graph analysis	*Report the dependent variable and connectivity measure, specifying weighted graph or binarized graph, subject- or group-level, and the global and/or node summaries used (e.g. clustering coefficient, efficiency, etc.).*
Multivariate modeling and predictive analysis	*Specify independent variables, features extraction and dimension reduction, model, training and evaluation metrics.*
